# House dust—a Pandora's box of antimicrobial resistance (AMR) activity?

**DOI:** 10.1093/sumbio/qvaf022

**Published:** 2025-09-16

**Authors:** David A Pearce, Matthew Crown, Andrew Nelson, Khadija Jabeen, Jonathan R Thompson, Ariadne Argyraki, Andrew S Hursthouse, Matthew Bashton, Jane A Entwistle

**Affiliations:** Faculty of Health and Life Sciences, Northumbria University, Newcastle upon Tyne NE1 8ST, UK; Faculty of Health and Life Sciences, Northumbria University, Newcastle upon Tyne NE1 8ST, UK; The Hub for Biotechnology in the Built Environment (HBBE), Faculty of Health and Life Sciences, Northumbria University, Newcastle upon Tyne, NE1 8ST, UK; Faculty of Health and Life Sciences, Northumbria University, Newcastle upon Tyne NE1 8ST, UK; Faculty of Engineering and Environment, Northumbria University, Newcastle upon Tyne NE1 8ST, UK; Faculty of Health and Life Sciences, Northumbria University, Newcastle upon Tyne NE1 8ST, UK; Department of Geology and Geoenvironment, National & Kapodistrian University of Athens, Panapistimiopolis, Zographou 157 84 Athens, Greece; School of Computing, Engineering & Physical Sciences, University of the West of Scotland, Paisley PA1 2BE Scotland, UK; Faculty of Health and Life Sciences, Northumbria University, Newcastle upon Tyne NE1 8ST, UK; The Hub for Biotechnology in the Built Environment (HBBE), Faculty of Health and Life Sciences, Northumbria University, Newcastle upon Tyne, NE1 8ST, UK; Faculty of Engineering and Environment, Northumbria University, Newcastle upon Tyne NE1 8ST, UK

## Abstract

The presence and spread of Antibiotic Resistant Bacteria (ARB) and Antibiotic Resistant Genes (ARGs) in the environment is now recognised as one of the top ten global public health threats to humanity. In a previous study, we used citizen science and MiSeq to target 16S rRNA gene amplicons to investigate house dust microbiomes across diverse households and found a core microbiome. In this study, we used shotgun metagenomics to target antimicrobial resistance (AMR) genes in order to investigate the potential for functional differences and to test the hypothesis that there was a core resistome associated with this core microbiome, including any patterns in a core resistome in terms of likely origin and mechanisms of action. In this study we did not find a core resistome, but found that the predominant and most diverse mechanisms of Anti-Microbial Resistance (AMR) in the dust samples were antibiotic target alteration and antibiotic efflux, accounting for ∼70% of cumulative RPKMs detected, potentially representing a compromise between the certainty of working and energy investment required. Despite the core home microbiome previously detected in diverse house dust samples, there was only limited evidence for a core resistome, with only two AMR genes present in all samples.

**Sustainability Statement**

Antimicrobial resistance (AMR) is one of the top global public health and development threats and is a truly global concern. Gaining a better understanding of the source, role and longevity of AMR genes in the domestic environment will enable us to develop better strategies to minimize its spread. As such this study feeds directly into UNSDG3 Good health and well-being.

## Introduction

Human activity pattern research from across Europe indicates that people spend approximately 56%–66% of their time at home and it is likely that this figure has increased due to the SARS-CoV-2 pandemic with increased adoption of hybrid working arrangements (Schweizer et al. 2007, Petrou et al. 2022). With approximately 70% of the world population projected to live in urban areas by 2050, there is now a growing need to better understand personal exposures sustained from a variety of substances in the built environment, specifically within the confines of individual or family's place of residence (United Nations, 2019). Environmental exposures have long played an important role in the dynamic balance between health and disease in humans and other multi-cellular organisms. House dust can form a significant component of everyday exposures within the domestic setting, contributing to the human exposome (Lanphear et al. 1996, Ding et al. 2020). This dust is a complex environmental matrix consisting of particles and fibres derived from a variety of sources including outdoor soil, vegetation, building materials (*e.g*. paint and furnishings), fabrics, human and animal dermis, small invertebrates and those originating from the use of disinfection, personal hygiene, cosmetics and indoor pest control products. It thus represents a highly heterogeneous and dynamic repository of physical, chemical, biological and microbiological materials found in the indoor environment that is also subject to routine changes in moisture, light and UV irradiation levels. The existence of localised chemical and physical gradients in this heterogeneous mixture and its continual interaction with living organisms indoors creates multiple niches for microbial colonization.

A typical household represents an environment that is relatively easy to access, not characterised by extremes in temperature, humidity or chemical gradients. Dust produced or contained within the home thus represents a persistent source of human exposure rendering it an ideal environmental medium in which inter-community interactions of microbes with each other and their physical environment may be examined, and clinically important relationships between these and their human hosts deduced.

The ecology of microbial life inside homes is increasingly being cited as being of significance to various aspects of human health and wellbeing (Dunn et al. 2013, National Academies of Sciences, Engineering, and Medicine 2017). It is now well-established that the human microbiome continually interacts with microorganisms in the surrounding environment, with the movement of species in both directions. As the human microbiome is essential for the health of the individual, understanding the nature, frequency and stability of this interaction, and the factors that govern it, is an important step towards increasing our collective understanding of how the management of our domestic environments can be improved to promote healthier lifestyles. Using the 16S rRNA amplicon sequencing approach, we showed a high degree of variability in the bacterial diversity of dust samples acquired from human dwellings (Thompson et al. 2021). There were, however, regional patterns in the biodiversity of the bacterial communities including evidence for a core house dust microbiome. What this study did not show was whether the functional diversity of these bacterial communities was consistent and whether consistent functional genes were present in the house dust samples. To test this hypothesis, we constructed a shotgun metagenomic library of a sub-set of the dust samples to specifically examine a key set of human health-related indicator functional genes—those coding for antimicrobial resistance (AMR).vacuum beyond the main home (e.g. in the garage, porch or veranda).

Infection by antibiotic-resistant bacteria is a major public health concern of the 21st century as the spread of AMR genes threatens to reduce the prevention and effective treatment of an array of infectious diseases (World Health Organisation 2014). Previous attempts to characterise antimicrobial resistance in the built environment have mainly been focused on public or semi-public spaces such as fitness and healthcare facilities or university campuses—environments that experience large footfall (Table 1). This study specifically investigated domestic settings adopting a citizen science approach, where the numbers of human receptors remained stable for extended periods of time and the occupant density was also low. To date, large numbers of diverse human dwellings have not been explored in this way, with existing studies focussing on multiple rooms or surfaces often confined to a single residence. In addition, swabbing appears to be the preferred method of dust collection for a range of indoor environments (Roberts et al. 2013, Kang et al. 2018, Cave et al. 2019).

**Table 1.** Previous studies of AMR in other indoor environments.

Study	Country	Sample type	Indoor environment	Most abundant ARGs
Roberts et al. 2013	USA (n = 379)	Swab	University buildings and student housing	*tetM, tetK, msrA, aadD, ermC*
Hartmann et al. 2016	USA (n = 44)	Vacuum	Multiple rooms/floor areas of single commercial building	*tetW, blaSRT-1, ermB*
Kang et al. 2018	Hong Kong (sample size unspecified)	Swab	Indoor surfaces of trains	*aph(3′), macB, ermC, msrA, tetK, ant4(4′)-Ia, tetM*
Cave et al. 2019	UK (n = 600)	Swab	Multiple surfaces/rooms in public areas and a single healthcare facility	*mecA, blaZ, qacA/B, dfrC, norA, ant(4′)-Ib, AAC(6′)-Ie-APH(2″)-Ia, fusB, msrA, ermC*,
Ben Maamar et al. 2020	USA (n = 166)	Vacuum	Multiple athletic and commercial buildings	*AAC(3)-VIIa, ErmO, CRP, AAC(3)-VIIIa, qacA, APH(3_)-Ia, vanRO, tetO, PC1_beta-lactamase_(blaZ), lnuA*
Luiken et al. 2022	Belgium, Bulgaria, Denmark, France, Germany, Italy, Netherlands, Poland and Spain (n = 947)	Electrostatic dustfall collectors	Poultry and pig farms	*tetW, ermB, aph(3′)-III, sul2*

In this study, we therefore employed a citizen science approach to investigate the house dust microbiomes of diverse households. We targeted antimicrobial resistance (AMR) genes in order to determine whether a core resistome was present, and to describe any potential patterns in a core resistome in terms of likely origin and mechanisms of action.

## Materials and methods

### Sample selection, handling & household meta-data collection

Samples consisted of composite dust samples collected from vacuum cleaner bags (either complete vacuum dust bags or sub-samples, such as where a bagless vacuum cleaner was used) acquired through a community science approach (Thompson et al. 2021). The samples were logged and stored, unopened, in the dark at room temperature. Participants were also required to complete a questionnaire (accessible at https://www.mapmyenvironment.com/homebiome/) as part of the Dustsafe/Home Biome community science study (Kourlaba et al. 2016). Of the vacuum dust samples available at that time, 19 were selected for this study to cover a broad range of household characteristics: these included samples from a national campaign within the UK, representing a regional spread across the UK (including Northern Ireland), and also samples from Greece specifically selected to provide additional heterogeneity and sourced from a different bioclimatic zone. Metadata provided by participants via the online survey revealed insights into the home and household characteristics and informed sample selection to ensure a diversity of characteristics (Table S1).

Samples were sieved to < 250 µm particle diameter in an Airstream® Generation 3 AC2 Class II Biological Safety Cabinet (ESCO^®^, Singapore) that had been sanitised with ethanol 70% v/v swabbing and exposure to UV radiation for 30 minutes prior to and between consecutive sieving processes. The mesh was utilised to remove larger particulates and fibrous material; and the sub-250 μm sieved fractions (commonly selected for the chemical analysis of dusts, sediments, and soils) were stored in 50 mL sterile falcon tubes until DNA extraction.

Trace metal analysis was also used as an independent verfication of sample heterogeneity. Approximately 4 g of the < 250 um dust was mixed with a wax binder and pressed into circular pellets. Trace metal concentrations were then determined using Energy Dispersive X-ray Fluorescence (ED-XRF) spectrometry. Instrument specifics, limits of detection (LoD), and quality control measures are reported in Isley et al. (2022).

### DNA extraction and sequencing

Isolation of microbial genomic DNA from each dust sample was performed using a Qiagen DNeasy 96 PowerSoil Pro Kit as per the manufacturers’ instructions. Extracted DNA samples were stored at −20°C until sequencing. Metagenomic library preparation was performed using Nextera XT DNA Library Preparation Kit as per manufacturer's instructions. Sequencing was performed by NU-OMICS using an Illumina NextSeq with a Mid-Output 300 cycle kit (150 bp paired end reads), to facilitate a sequencing depth of 10 M reads per sample.

#### Bioinformatic analysis

Analysis was performed using a Snakemake pipeline (Krueger et al. 2021) (available at: https://github.com/m-crown/HomeBiomeAMR). Briefly, samples were quality trimmed and adapters removed using Trim Galore (Bushnell 2014). Human reads were filtered with BBMap v38.87 and a custom-masked human genome (Lu and Salzberg 2020). Reads were classified using Kraken2 and the PlusPFP database (Kaminski et al. 2015). Reads classified as bacterial were extracted and used as input for ShortBRED v0.9.5 analysis (Lu et al. 2017). Bracken v2.9 (Vill 2023) was used to estimate the abundance of bacterial species in the samples, and abundance at various levels was visualised using the bracken_plot tool (configured to use mean abundance instead of median abundance) (Alcock et al. 2020).

A custom ShortBRED database was created with ShortBRED-identify, a tool for creating custom marker databases and quantifying microbial protein families in metagenomic data, using the Comprehensive Antibiotic Resistance Database (CARD v3.2.8, October 2023 release), a manually curated collection of known antibiotic resistance genes and the UniRef90 (release 2023–09-13) database a comprehensive database of non-redundant protein sequences, clustering UniProtKB sequences at the 90% identity level, as a background (Suzek et al. 2015; The R project for statistical computing 2023).

The preprocessing steps for building the database are described in the shortbred_build.txt of the GitHub repo. Each sample was analysed with ShortBRED-quantify to identify antibiotic resistance genes (ARGs). Individual ShortBRED reports were merged and further analyzed in R, to categorise identified ARGs into different drug classes and resistance mechanisms based on CARD ontology (Ondov et al. 2011). The data were then transformed into a format compatible with the Krona visualization tool, an interactive metagenomic visualization tool that allows the exploration of hierarchical data like antibiotic resistance ontologies (Ding et al. 2020). This provided an interactive exploration of antibiotic resistance profiles in the samples.

## Results

### Sample selection

Across the samples selected, we ensured a spread of home ages; 9 newer builds (<50 years of age), 7 aged 51–100 years, and 3 older (>100 years of age). Three common property types were also represented (i.e. detached, semi-detached and flat), however nearly all properties were made of brick construction. A range of dominant flooring types and primary heating sources were represented in the selected sub-samples; carpet and wood flooring were equally represented (8 homes each) with 3 homes tiled, whilst the primary source of heating was natural gas (12 homes), but with oil, wood, electric and other also represented (4 homes, 1, 1, and 1 home, respectively).

In terms of the occupants themselves, households from 1 up to 6 people were represented, with most households having both male and female genders present, and 5 out of the 19 households having at least one pet. Four households contained regular indoor smokers, most vacuumed at least weekly, with 8 households also using their vacuum beyond the main home (e.g. in the garage, porch or veranda).

Seven trace elements (Arsenic (As), chromium (Cr), copper (Cu), manganese (Mn), nickel (Ni), lead (Pb) and zinc (Zn)), were selected for analysis as they are known to occur in potentially toxic concentrations in residential environments. Descriptive statistics highlight the order of magnitude concentration range across each of the trace elements (with the exception of Mn) across the 19 samples (Table S2).

### Sample QC and read classification

**Figure 1.**
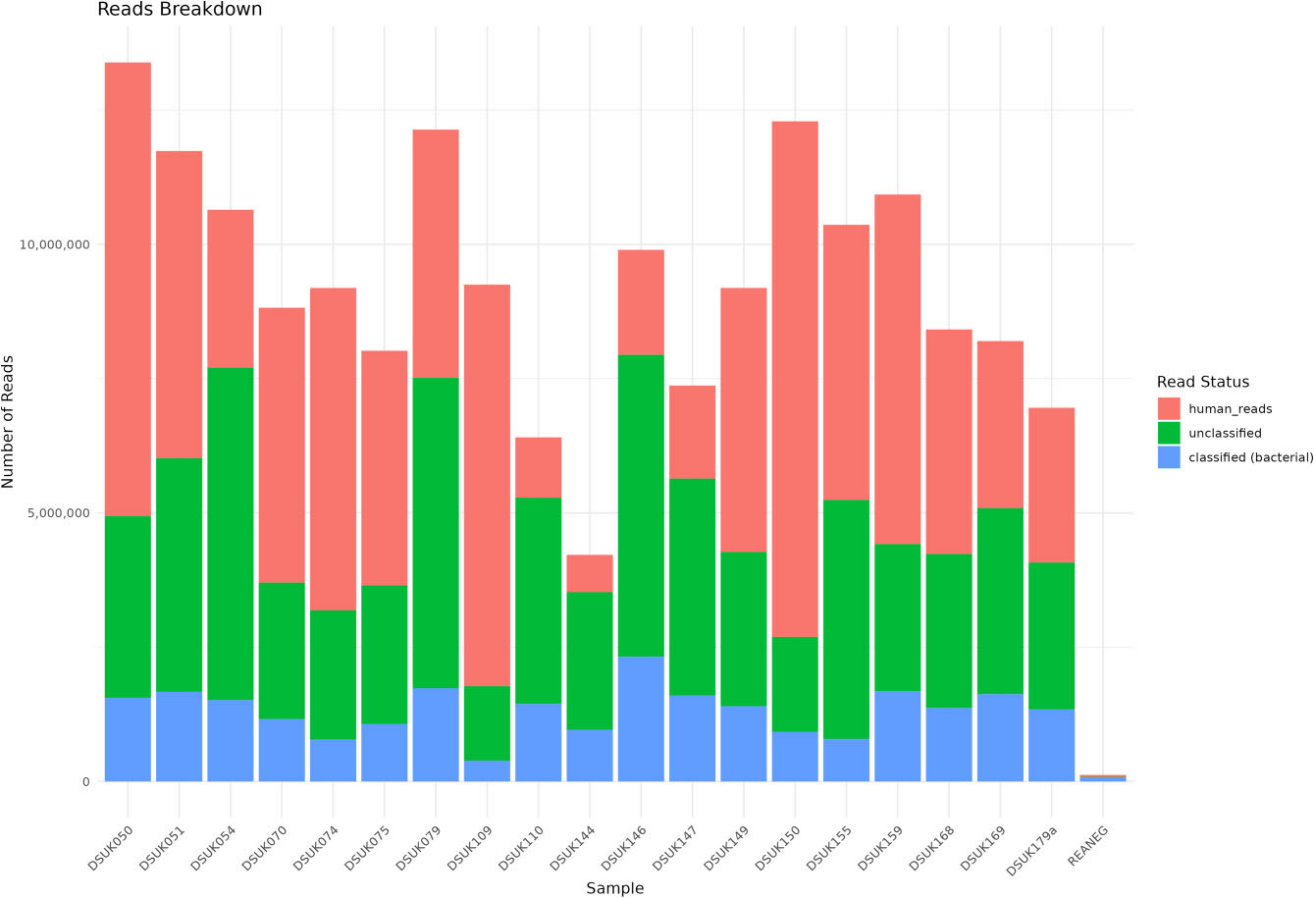
Reads retained at each step of sample QC and processing

A total of 19 samples were analysed with an average raw sample depth of 9.34 million reads, ranging from 4.22 to 13.39 million reads (Table S3). The average sample depth after pre- processing was 4.78 million reads, with a minimum of 1.77 million and a maximum of 7.94 million reads. Average sample depth of bacterial reads was 1.33 million. An average of 15% of reads were retained following sample pre-processing and classification for input to downstream AMR analysis (Fig. 1).

### Bacterial species present

**Figure 2.**
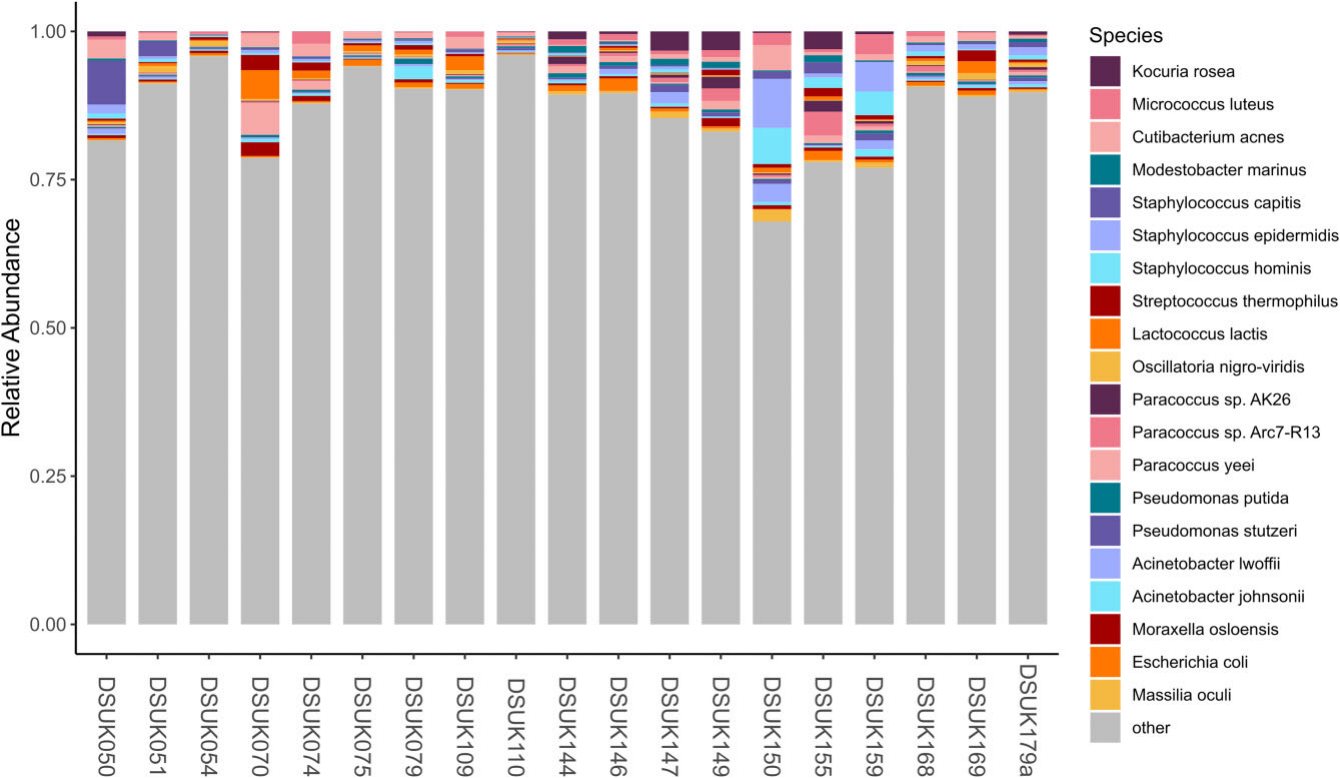
Relative abundance of top 20 species identified in house dust from homes in UK and Greece. The “Other” category comprises all those species identified outside of the top 20 species by mean relative abundance

**Figure 3.**
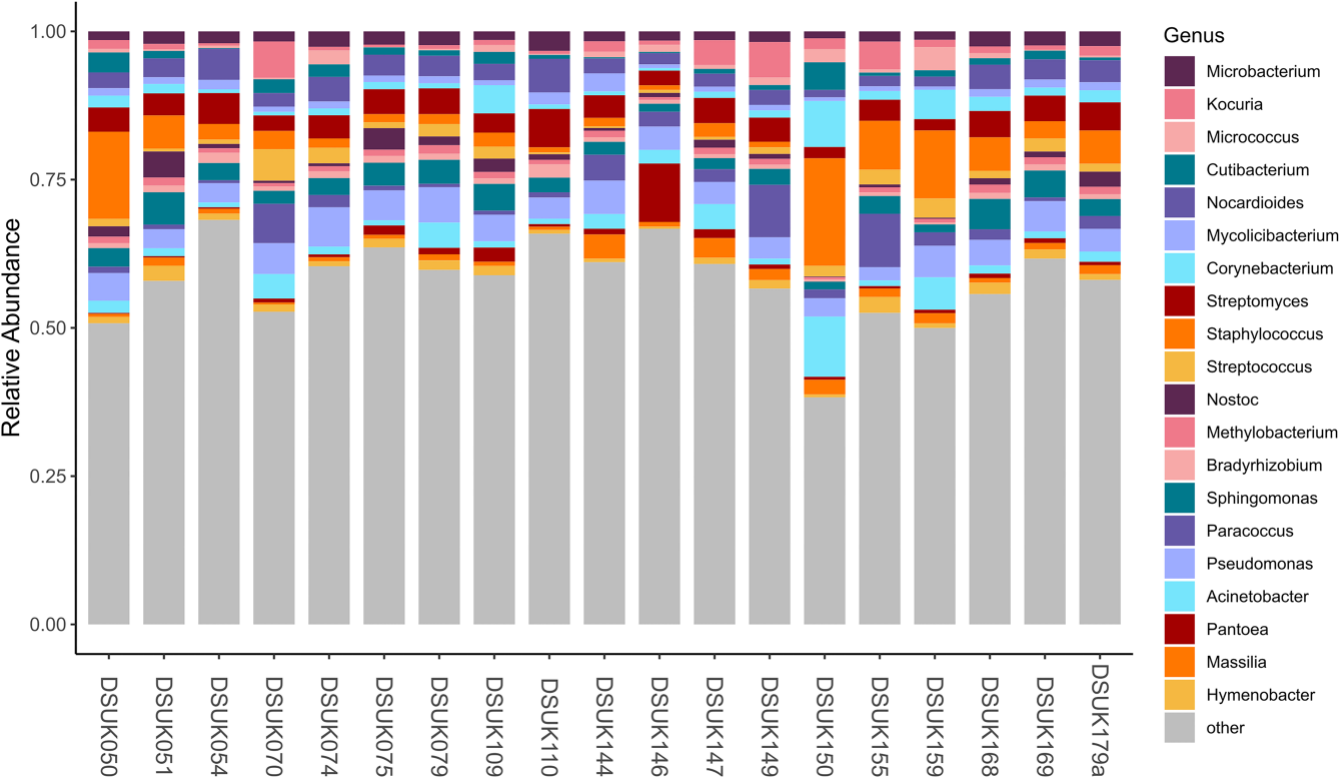
Relative abundance of top 20 genera identified in house dust from homes in UK and Greece. The “Other” category comprises all those genera identified outside of the top 20 genera by mean relative abundance

From the DNA sequence data obtained, it was possible to identify 6 189 bacteria. The first ∼2 500 of the most abundant sequences were identified in all 19 samples, the next ∼3 350 species were identified in the majority of the samples and the remaining 343 only occurred in one or two samples. The top 50 species identified (in order of frequency) are listed in Table 2. Highly abundant species (>1% mean relative abundance across all samples) were *Cutibacterium acnes* and *Staphylococcus epidermidis*. The frequency distribution of the top 20 species and genera are given in Figs. 2 and 3 respectively.

**Table 2.** Top 50 Species Table

Species	Me an	DSUK 050	DSUK 051	DSUK 054	DSUK 070	DSUK 074	DSUK 075	DSUK 079	DSUK 109	DSUK 110	DSUK 144	DSUK 146	DSUK 147	DSUK 149	DSUK 150	DSUK 155	DSUK 159	DSUK 168	DSUK 169	DSUK 179a
*Cutibacterium acnes*	1.27	3.14	1.18	0.10	2.31	2.07	1.11	0.89	1.98	0.62	0.22	0.21	0.77	0.76	4.27	0.54	1.04	1.04	1.40	0.43
*Staphylococcus epidermidis*	1.17	1.48	0.46	0.10	0.48	0.41	0.35	0.40	0.22	0.07	0.21	0.17	0.46	0.18	8.28	0.68	4.98	1.07	0.84	1.33
*Staphylococcus hominis*	0.90	0.87	0.51	0.14	0.35	0.22	0.10	0.32	0.05	0.08	0.25	0.07	0.60	0.06	6.09	1.74	3.99	0.75	0.20	0.73
*Staphylococcus capitis*	0.89	7.43	2.60	0.07	0.29	0.33	0.16	0.25	0.48	0.03	0.04	0.21	0.23	0.06	1.21	1.92	0.08	0.26	0.29	0.90
*Lactococcus lactis*	0.86	0.23	0.46	0.22	4.87	1.29	0.96	0.81	2.34	0.22	0.07	0.44	0.05	0.09	0.78	0.61	0.19	0.46	2.00	0.28
*Micrococcus luteus*	0.82	0.58	0.23	0.41	0.22	2.06	0.08	0.22	0.91	0.14	0.88	1.03	0.58	1.20	2.02	0.48	3.38	0.77	0.15	0.26
*Paracoccus yeei*	0.78	0.16	0.16	0.07	5.39	1.45	0.23	0.17	0.09	0.11	1.14	0.97	0.25	1.43	0.33	1.27	0.61	0.23	0.13	0.55
*Kocuria rosea*	0.75	0.85	0.10	0.04	0.15	0.06	0.04	0.05	0.04	0.10	1.36	0.46	3.28	3.16	0.29	3.02	0.48	0.07	0.09	0.55
*Streptococcus thermophilus*	0.72	0.35	0.20	0.48	2.56	1.39	0.38	0.78	0.42	0.15	0.06	0.32	0.09	0.94	0.64	1.44	0.69	0.43	1.86	0.40
*Escherichia coli*	0.63	0.29	0.19	0.38	0.27	0.26	1.08	0.76	0.83	0.24	0.97	2.12	0.50	0.35	0.13	1.54	0.46	0.62	0.76	0.17
*Paracoccus* sp. Arc7- R13	0.60	0.13	0.13	0.10	0.20	0.16	0.08	0.08	0.15	0.28	0.39	0.55	0.73	2.13	0.34	4.03	0.52	0.90	0.10	0.40
*Acinetobacter lwoffii*	0.60	0.85	0.37	0.09	0.23	0.11	0.08	0.34	0.25	0.22	0.25	0.84	1.90	0.14	3.06	0.10	1.50	0.38	0.10	0.51
*Moraxella osloensis*	0.55	0.50	0.36	0.41	2.29	0.88	0.13	0.55	0.17	0.07	0.36	0.37	0.31	1.41	0.65	0.57	0.53	0.21	0.44	0.30
*Acinetobacter johnsonii*	0.49	0.23	0.13	0.18	0.53	0.41	0.17	2.17	0.41	0.21	0.46	0.38	0.57	0.17	0.53	0.26	1.24	0.31	0.47	0.52
*Paracoccus* sp. AK26	0.42	0.14	0.05	0.04	0.15	0.09	0.08	0.06	0.12	0.10	1.25	0.27	0.53	1.96	0.25	1.87	0.45	0.16	0.06	0.44
*Oscillatoria nigro- viridis*	0.42	0.38	1.13	0.81	0.33	0.21	0.32	0.38	0.67	0.45	0.05	0.17	0.18	0.17	0.16	0.11	0.12	0.67	1.10	0.60
*Massilia oculi*	0.40	0.09	0.15	0.13	0.05	0.16	0.06	0.25	0.12	0.08	0.56	0.36	1.09	0.50	1.97	0.34	0.84	0.16	0.24	0.38
*Modestobacter marinus*	0.39	0.36	0.10	0.10	0.17	0.09	0.15	0.20	0.12	0.15	1.13	0.30	1.06	1.03	0.20	1.18	0.19	0.20	0.22	0.54
*Pseudomonas putida*	0.38	0.20	0.19	0.21	0.41	0.32	0.24	0.90	0.26	0.29	0.76	0.64	0.21	0.48	0.20	0.22	0.47	0.43	0.42	0.37
*Pseudomonas stutzeri*	0.37	0.11	0.13	0.11	0.08	0.19	0.09	0.09	0.17	0.37	0.25	0.52	1.22	0.67	0.65	0.15	1.21	0.22	0.11	0.62
*Pseudomonas aeruginosa*	0.35	0.20	0.21	0.92	0.38	0.97	0.20	0.28	0.25	0.34	0.23	0.17	0.22	0.33	0.22	0.17	0.34	0.75	0.25	0.22
*Friedmanniella luteola*	0.35	0.13	0.26	0.16	0.33	0.31	0.45	0.43	0.35	0.34	0.35	0.12	0.36	0.68	0.15	0.68	0.16	0.38	0.39	0.59
*Geodermatophilus obscurus*	0.34	0.40	0.08	0.08	0.11	0.07	0.11	0.15	0.09	0.12	1.06	0.32	0.96	0.91	0.22	0.92	0.20	0.14	0.18	0.38
*Friedmanniella sagamiharensis*	0.34	0.18	0.29	0.21	0.40	0.44	0.46	0.57	0.44	0.37	0.19	0.07	0.28	0.31	0.12	0.55	0.16	0.52	0.36	0.49
*Acinetobacter schindleri*	0.33	0.03	0.03	0.02	0.04	0.03	0.01	0.09	0.02	0.02	0.26	0.14	0.42	0.09	4.73	0.03	0.28	0.03	0.02	0.06
*Pantoea agglomerans*	0.33	0.07	0.14	0.12	0.27	0.14	0.69	0.43	0.40	0.15	0.29	1.16	0.24	0.37	0.24	0.17	0.15	0.51	0.64	0.16
*Blastococcus saxobsidens*	0.33	0.51	0.07	0.07	0.10	0.07	0.10	0.14	0.08	0.11	1.27	0.31	0.92	0.78	0.22	0.63	0.18	0.13	0.18	0.47
*Kocuria palustris*	0.31	0.27	0.11	0.12	0.37	0.24	0.11	0.21	0.15	0.15	0.16	0.11	0.23	0.64	1.11	0.13	0.43	0.75	0.33	0.33
*Salmonella enterica*	0.28	0.08	0.41	0.25	0.13	0.13	0.23	0.20	0.53	0.18	0.37	1.22	0.46	0.08	0.08	0.20	0.30	0.11	0.32	0.12
*Stenotrophomonas maltophilia*	0.28	0.15	0.16	0.18	0.27	0.22	0.21	0.24	0.11	0.16	0.70	0.78	0.22	0.13	0.65	0.11	0.29	0.15	0.33	0.21
*Klebsiella pneumoniae*	0.26	0.06	0.06	0.08	0.23	0.07	0.15	0.72	0.48	0.06	0.23	1.54	0.35	0.12	0.06	0.05	0.19	0.16	0.22	0.11
*Staphylococcus pettenkoferi*	0.26	0.92	0.49	0.00	0.02	0.03	0.02	0.15	0.26	0.00	0.00	0.02	0.05	0.01	0.34	1.87	0.22	0.03	0.06	0.42
*Paenibacillus larvae*	0.26	0.16	0.28	0.60	0.07	0.13	0.11	0.38	0.47	0.76	0.26	0.07	0.32	0.16	0.04	0.36	0.05	0.18	0.24	0.26
*Enterobacter hormaechei*	0.25	0.01	0.03	0.06	0.41	0.04	0.06	0.04	0.13	0.07	0.43	1.64	0.22	0.07	0.17	0.04	0.95	0.10	0.15	0.13
*Staphylococcus cohnii*	0.25	1.53	0.45	0.03	0.27	0.03	0.15	0.04	0.04	0.02	0.05	0.01	0.11	0.08	0.32	0.42	0.81	0.26	0.05	0.02
*Lactobacillus iners*	0.24	0.01	0.02	0.02	0.00	1.42	0.01	0.41	0.00	0.16	0.00	0.00	0.04	0.12	0.87	0.00	0.44	0.19	0.00	0.78
*Nocardioides* sp. CF8	0.23	0.20	0.26	0.50	0.19	0.38	0.30	0.24	0.24	0.46	0.13	0.10	0.13	0.12	0.08	0.10	0.11	0.33	0.24	0.26
*Pseudomonas fluorescens*	0.23	0.43	0.22	0.15	0.34	0.44	0.40	0.33	0.34	0.19	0.13	0.07	0.07	0.09	0.07	0.15	0.08	0.37	0.25	0.18
*Streptococcus mitis*	0.22	0.12	0.02	0.03	0.86	0.36	0.07	0.41	0.31	0.02	0.05	0.03	0.07	0.02	0.25	0.27	0.86	0.21	0.05	0.23
*Serratia marcescens*	0.22	0.08	0.03	0.05	0.15	0.05	0.04	1.35	1.12	0.03	0.04	0.38	0.07	0.03	0.02	0.02	0.11	0.35	0.13	0.05
*Staphylococcus warneri*	0.21	0.27	0.13	0.11	0.41	0.11	0.10	0.04	0.07	0.07	0.19	0.08	0.05	0.03	0.22	0.07	0.15	0.82	0.46	0.59
*Pseudonocardia* sp. Gen01	0.20	0.43	0.16	0.18	0.10	0.10	0.27	0.23	0.16	0.22	0.27	0.11	0.25	0.14	0.08	0.18	0.07	0.23	0.27	0.31
*Kocuria varians*	0.19	0.10	0.09	0.06	1.86	0.13	0.06	0.19	0.16	0.05	0.03	0.02	0.03	0.21	0.09	0.10	0.07	0.07	0.12	0.23
*Sphingomonas* sp. PAMC26645	0.19	0.22	0.39	0.20	0.14	0.23	0.22	0.34	0.25	0.13	0.05	0.03	0.06	0.08	0.04	0.11	0.04	0.54	0.45	0.12
*Pantoea vagans*	0.19	0.02	0.03	0.03	0.13	0.03	0.15	0.16	0.46	0.05	0.12	1.85	0.26	0.03	0.05	0.02	0.07	0.04	0.04	0.03
*Nocardioides* sp. S- 1144	0.18	0.19	0.26	0.34	0.15	0.26	0.22	0.21	0.17	0.31	0.12	0.08	0.12	0.11	0.06	0.10	0.08	0.28	0.23	0.20
*Corynebacterium segmentosum*	0.18	0.23	0.11	0.02	0.06	0.10	0.13	0.05	0.18	0.01	0.04	0.03	0.11	0.07	1.04	0.14	0.59	0.22	0.15	0.20
*Erwinia billingiae*	0.18	0.07	0.14	0.09	0.42	0.06	1.72	0.26	0.11	0.10	0.01	0.09	0.02	0.01	0.02	0.02	0.02	0.11	0.17	0.02
*Staphylococcus aureus*	0.18	0.43	0.18	0.08	0.08	0.05	0.05	0.15	0.56	0.17	0.06	0.03	0.06	0.06	0.35	0.40	0.21	0.25	0.06	0.16
*Acinetobacter ursingii*	0.17	0.04	0.03	0.09	1.89	0.19	0.05	0.18	0.02	0.01	0.08	0.04	0.04	0.02	0.18	0.01	0.36	0.02	0.04	0.03

### AMR gene overall diversity

**Figure 4.**
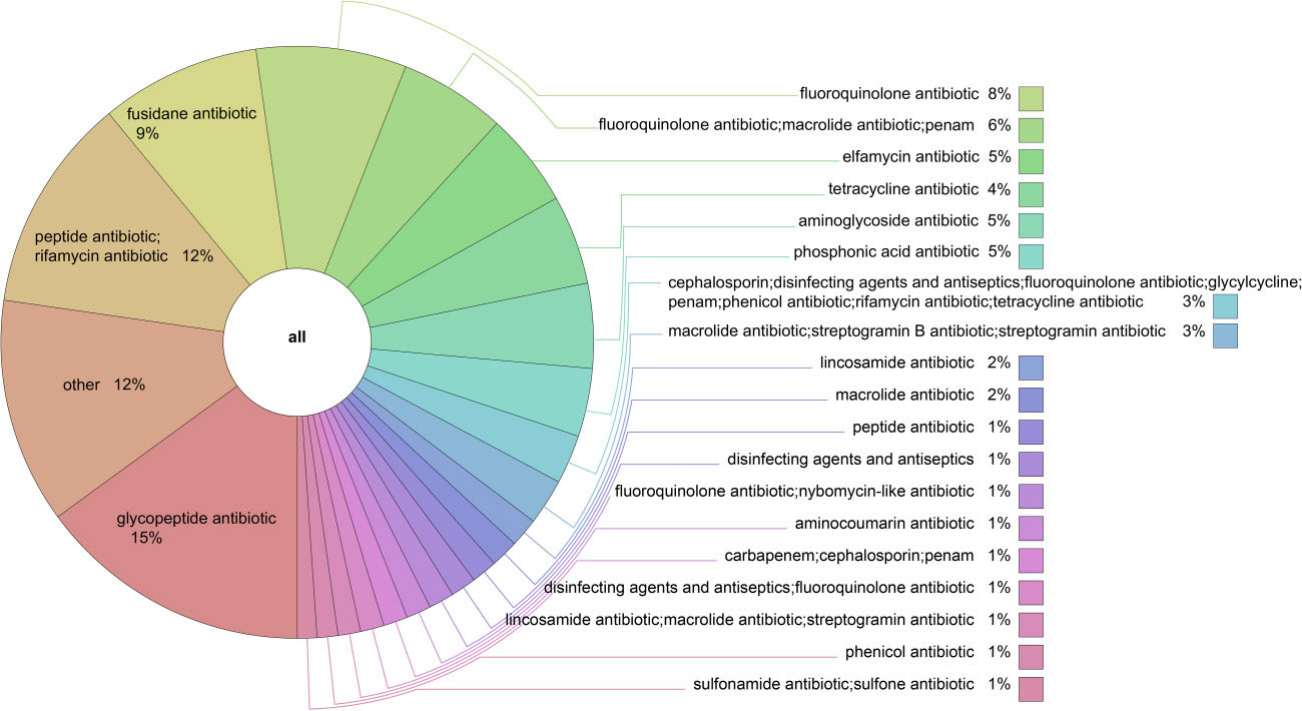
Krona plot showing the distribution of AMR drug classes across the combined sample set. AMR genes were not equally distributed across the different antibiotic groups, with Fusidane, Peptide antibiotic, rifamycin antibiotic, tetracycline and glycopeptide antibiotic the predominant classes.

The cumulative RPKM abundance across all detected AMR genes was 15 817, spread across 182 AMR genes in 80 gene families (Table 3) and 70 drug classes (Table 4). The five most abundance AMR genes accounted for ∼45% of the total RPKMs detected, with 17 AMR genes present at > 1% cumulative RPKM. The specific distribution of drug classes among samples is given in the Krona plot in Fig. 4. The top drug classes present were to glycopeptide antibiotic (15%), peptide antibiotic; rifamycin antibiotic (12%), fusidane antibiotic ∼9%), fluoroquinolone antibiotic (∼8%) and fluoroquinolone antibiotic; macrolide antibiotic; penam (∼6%) (Table 4). Elfamycin resistant *EF-Tu* and and glycopeptide resistance gene cluster; *vanR* were detected in all samples and were the first and fourth highest abundance AMR genes detected across all samples (percentage of total RPKMs detected 25.66 and 8.84% respectively). Additionally, Major facilitator Superfamily (MFS) antibiotic efflux pump was found in all but one sample and Erm 23S ribosomal RNA methyltransferase all but two.

**Table 3.** AMR Gene Family Table.

AMR gene family	No. samples	% samples	Total RPKMs (all samples)	% RPKMSs (all samples)
elfamycin resistant *EF-Tu*	19	100.00	815.20	8.84
glycopeptide resistance gene cluster;*vanR*	19	100.00	2367.27	25.66
resistance-nodulation-cell division (RND) antibiotic efflux pump	19	100.00	62.14	0.67
tetracycline-resistant ribosomal protection protein	19	100.00	55.21	0.60
major facilitator superfamily (MFS) antibiotic efflux pump	18	94.74	71.68	0.78
Erm 23S ribosomal RNA methyltransferase	17	89.47	43.76	0.47
*msr*-type ABC-F protein	14	73.68	205.93	2.23
ATP-binding cassette (ABC) antibiotic efflux pump	13	68.42	37.50	0.41
Target protecting FusB-type protein conferring resistance to Fusidic acid	13	68.42	39.55	0.43
fluoroquinolone resistant *gyrB*	13	68.42	89.69	0.97
fluoroquinolone resistant *gyrA*	12	63.16	54.66	0.59
macrolide phosphotransferase (MPH)	11	57.89	116.66	1.26
antibiotic-resistant rpsL	10	52.63	518.68	5.62
small multidrug resistance (SMR) antibiotic efflux pump	10	52.63	23.15	0.25
ATP-binding cassette (ABC) antibiotic efflux pump;General Bacterial Porin with reduced permeability to beta-lactams;major facilitator superfamily (MFS) antibiotic efflux pump;resistance-nodulation-cell division (RND) antibiotic efflux pump	9	47.37	100.25	1.09
antibiotic-resistant *UhpT*	9	47.37	101.98	1.11
rifamycin-resistant beta-subunit of RNA polymerase (*rpoB*)	9	47.37	930.77	10.09
trimethoprim resistant dihydrofolate reductase *dfr*	9	47.37	133.85	1.45
antibiotic resistant *fusA*	8	42.11	1236.21	13.40
fosfomycin thiol transferase	8	42.11	16.66	0.18
aminocoumarin resistant *gyrB*	7	36.84	106.35	1.15
antibiotic-resistant *ptsI* phosphotransferase	7	36.84	172.52	1.87
class A Mycobacterium abscessus beta-lactamase	7	36.84	23.65	0.26
*ANT(3′')*	6	31.58	16.80	0.18
antibiotic resistant *fabG*	6	31.58	128.39	1.39
*vga*-type ABC-F protein	6	31.58	94.14	1.02
ATP-binding cassette (ABC) antibiotic efflux pump;major facilitator superfamily (MFS) antibiotic efflux pump;resistance-nodulation-cell division (RND) antibiotic efflux pump	5	26.32	182.38	1.98
*BlaZ* beta-lactamase	5	26.32	41.14	0.45
*OXA* beta-lactamase	5	26.32	69.91	0.76
lincosamide nucleotidyltransferase (*LNU*)	5	26.32	79.77	0.86
non-erm 23S ribosomal RNA methyltransferase (G748)	5	26.32	44.29	0.48
CARB beta-lactamase	4	21.05	5.44	0.06
antibiotic resistant ATP synthase	4	21.05	9.58	0.10
antibiotic-resistant *murA* transferase	4	21.05	31.43	0.34
fluoroquinolone resistant *parE*	4	21.05	204.33	2.21
methicillin resistant *PBP2*	4	21.05	25.53	0.28
*pmr* phosphoethanolamine transferase	4	21.05	21.96	0.24
sulfonamide resistant dihydropteroate synthase *folP*	4	21.05	96.60	1.05
sulfonamide resistant *sul*	4	21.05	24.92	0.27
antibiotic resistant *fabI*	3	15.79	83.67	0.91
chloramphenicol acetyltransferase (*CAT*)	3	15.79	17.61	0.19
ACT beta-lactamase	2	10.53	17.10	0.19
*ANT(4′)*	2	10.53	12.67	0.14
*APH(6)*	2	10.53	4.41	0.05
Acinetobacter mutant *Lpx* gene conferring resistance to colistin	2	10.53	8.91	0.10
General Bacterial Porin with reduced permeability to beta-lactams;resistance-nodulation-cell division (RND) antibiotic efflux pump	2	10.53	20.19	0.22
*MOX* beta-lactamase	2	10.53	10.75	0.12
Penicillin-binding protein mutations conferring resistance to beta-lactam antibiotics	2	10.53	34.23	0.37
*TEM* beta-lactamase	2	10.53	98.06	1.06
daptomycin resistant *pgsA*	2	10.53	17.18	0.19
fluoroquinolone resistant *parC*	2	10.53	33.67	0.36
major facilitator superfamily (MFS) antibiotic efflux pump;resistance-nodulation-cell division (RND) antibiotic efflux pump	2	10.53	30.98	0.34
multidrug and toxic compound extrusion (MATE) transporter	2	10.53	6.44	0.07
streptothricin acetyltransferase (*SAT*)	2	10.53	2.43	0.03
undecaprenyl pyrophosphate related proteins	2	10.53	40.21	0.44
*AAC(6′)*	1	5.26	35.62	0.39
*ANT(6)*	1	5.26	17.29	0.19
*APH(3′')*	1	5.26	27.40	0.30
*APH(3′)*	1	5.26	8.74	0.09
CMH beta-lactamase	1	5.26	6.24	0.07
General Bacterial Porin with reduced permeability to beta-lactams	1	5.26	35.98	0.39
Intrinsic peptide antibiotic resistant *Lps*	1	5.26	18.52	0.20
*MCR* phosphoethanolamine transferase	1	5.26	4.17	0.05
*MipA*-interacting Protein	1	5.26	44.30	0.48
*PER* beta-lactamase	1	5.26	5.43	0.06
*RbpA* bacterial RNA polymerase-binding protein	1	5.26	3.33	0.04
Sugar Porin (SP)	1	5.26	23.98	0.26
ampC-type beta-lactamase	1	5.26	5.05	0.05
antibiotic resistant *fusE*	1	5.26	25.33	0.27
antibiotic resistant *nfsA*	1	5.26	25.17	0.27
antibiotic-resistant *GlpT*	1	5.26	3.35	0.04
antibiotic-resistant *cya* adenylate cyclase	1	5.26	29.97	0.32
antibiotic-resistant isoleucyl-tRNA synthetase (*ileS*)	1	5.26	2.32	0.03
daptomycin resistant *liaF*	1	5.26	5.76	0.06
daptomycin resistant *walK*	1	5.26	33.12	0.36
intrinsic colistin resistant phosphoethanolamine transferase	1	5.26	6.27	0.07
*kdpDE*	1	5.26	8.69	0.09
quinolone resistance protein (*qnr*)	1	5.26	2.89	0.03
spectinomycin resistant *rpsE*	1	5.26	4.75	0.05
tetracycline inactivation enzyme	1	5.26	4.27	0.05

**Table 4.** AMR Drug Class Table.

Drug class	Total RPKMs (all samples)	% RPKMs (all samples)
glycopeptide antibiotic	2367.27	14.97
peptide antibiotic;rifamycin antibiotic	1861.54	11.77
fusidane antibiotic	1380.20	8.73
fluoroquinolone antibiotic	1295.41	8.19
fluoroquinolone antibiotic;macrolide antibiotic;penam	917.92	5.80
elfamycin antibiotic	815.20	5.15
aminoglycoside antibiotic	770.70	4.87
phosphonic acid antibiotic	731.65	4.63
tetracycline antibiotic	588.14	3.72
cephalosporin;disinfecting agents and antiseptics;fluoroquinolone antibiotic;glycylcycline;penam;phenicol antibiotic;rifamycin antibiotic;tetracycline antibiotic	423.17	2.68
macrolide antibiotic;streptogramin B antibiotic;streptogramin antibiotic	397.72	2.51
lincosamide antibiotic	249.05	1.57
macrolide antibiotic	239.91	1.52
peptide antibiotic	223.08	1.41
disinfecting agents and antiseptics	220.25	1.39
fluoroquinolone antibiotic;nybomycin-like antibiotic	218.62	1.38
aminocoumarin antibiotic	212.71	1.34
carbapenem;cephalosporin;penam	209.73	1.33
disinfecting agents and antiseptics;fluoroquinolone antibiotic	204.66	1.29
lincosamide antibiotic;macrolide antibiotic;streptogramin antibiotic	194.38	1.23
phenicol antibiotic	179.68	1.14
sulfonamide antibiotic;sulfone antibiotic	169.10	1.07
aminocoumarin antibiotic;aminoglycoside antibiotic	150.91	0.95
carbapenem;cephalosporin;cephamycin;monobactam;penam	145.05	0.92
diaminopyrimidine antibiotic	133.85	0.85
carbapenem;cephalosporin;cephamycin;disinfecting agents and antiseptics;fluoroquinolone antibiotic;glycylcycline;monobactam;penam;penem;phenicol antibiotic;rifamycin antibiotic;tetracycline antibiotic	120.44	0.76
diaminopyrimidine antibiotic;fluoroquinolone antibiotic;phenicol antibiotic	114.99	0.73
lincosamide antibiotic;macrolide antibiotic;streptogramin A antibiotic;streptogramin B antibiotic;streptogramin antibiotic	111.94	0.71
aminocoumarin antibiotic;aminoglycoside antibiotic;carbapenem;cephalosporin;cephamycin;disinfecting agents and antiseptics;fluoroquinolone antibiotic;glycylcycline;macrolide antibiotic;penam;penem;peptide antibiotic;phenicol antibiotic;rifamycin antibiotic;tetracycline antibiotic	99.64	0.63
cephalosporin;monobactam;penam;penem	98.06	0.62
lincosamide antibiotic;pleuromutilin antibiotic;streptogramin A antibiotic;streptogramin antibiotic	94.14	0.60
aminocoumarin antibiotic;fluoroquinolone antibiotic	89.69	0.57
disinfecting agents and antiseptics;isoniazid-like antibiotic	83.67	0.53
disinfecting agents and antiseptics;nucleoside antibiotic	78.40	0.50
lincosamide antibiotic;macrolide antibiotic	55.21	0.35
penam	52.02	0.33
fluoroquinolone antibiotic;macrolide antibiotic;penam;tetracycline antibiotic	51.87	0.33
cephalosporin;cephamycin;fluoroquinolone antibiotic;penam	47.81	0.30
aminoglycoside antibiotic;fluoroquinolone antibiotic	44.30	0.28
carbapenem;cephalosporin;cephamycin;monobactam;penam;penem	35.98	0.23
diaminopyrimidine antibiotic;fluoroquinolone antibiotic;glycylcycline;nitrofuran antibiotic;tetracycline antibiotic	31.17	0.20
nitroimidazole antibiotic	28.79	0.18
nitrofuran antibiotic	25.17	0.16
cephalosporin;disinfecting agents and antiseptics;fluoroquinolone antibiotic;glycopeptide antibiotic;glycylcycline;oxazolidinone antibiotic;penam;phenicol antibiotic;rifamycin antibiotic;tetracycline antibiotic	25.11	0.16
fluoroquinolone antibiotic;tetracycline antibiotic	23.98	0.15
cephalosporin;penam;penem	23.65	0.15
aminoglycoside antibiotic;cephalosporin;cephamycin;penam	20.49	0.13
cephalosporin;cephamycin;disinfecting agents and antiseptics;fluoroquinolone antibiotic;glycylcycline;penam;phenicol antibiotic;rifamycin antibiotic;tetracycline antibiotic	19.21	0.12
carbapenem;cephalosporin;cephamycin;penam	17.10	0.11
macrolide antibiotic;streptogramin antibiotic	14.13	0.09
cephalosporin;cephamycin;penam	10.75	0.07
cephalosporin;cephamycin;fluoroquinolone antibiotic;macrolide antibiotic;penam;tetracycline antibiotic	10.09	0.06
diarylquinoline antibiotic	9.58	0.06
disinfecting agents and antiseptics;macrolide antibiotic;tetracycline antibiotic	8.87	0.06
disinfecting agents and antiseptics;fluoroquinolone antibiotic;lincosamide antibiotic;nucleoside antibiotic;phenicol antibiotic	7.45	0.05
aminocoumarin antibiotic;aminoglycoside antibiotic;carbapenem;cephalosporin;cephamycin;diaminopyrimidine antibiotic;fluoroquinolone antibiotic;macrolide antibiotic;monobactam;penam;penem;peptide antibiotic;phenicol antibiotic;sulfonamide antibiotic;tetracycline antibiotic	7.30	0.05
aminocoumarin antibiotic;carbapenem;cephalosporin;cephamycin;diaminopyrimidine antibiotic;fluoroquinolone antibiotic;macrolide antibiotic;monobactam;penam;penem;peptide antibiotic;phenicol antibiotic;sulfonamide antibiotic;tetracycline antibiotic	6.63	0.04
glycylcycline;tetracycline antibiotic	6.44	0.04
cephalosporin	6.24	0.04
carbapenem;cephalosporin;monobactam;penam;penem	5.43	0.03
cephalosporin;penam	5.05	0.03
nucleoside antibiotic	4.85	0.03
lincosamide antibiotic;nucleoside antibiotic	4.52	0.03
rifamycin antibiotic	3.33	0.02
carbapenem;cephalosporin;diaminopyrimidine antibiotic;fluoroquinolone antibiotic;lincosamide antibiotic;macrolide antibiotic;penem;phenicol antibiotic;rifamycin antibiotic;tetracycline antibiotic	3.12	0.02
aminoglycoside antibiotic;fluoroquinolone antibiotic;phosphonic acid antibiotic	2.33	0.01
mupirocin-like antibiotic	2.32	0.01
sulfonamide antibiotic	2.25	0.01
antibacterial free fatty acids	2.06	0.01
aminoglycoside antibiotic;carbapenem;cephalosporin;fluoroquinolone antibiotic;macrolide antibiotic;penam;penem;peptide antibiotic	1.43	0.01

### AMR gene frequency by mode of activity

**Figure 5.**
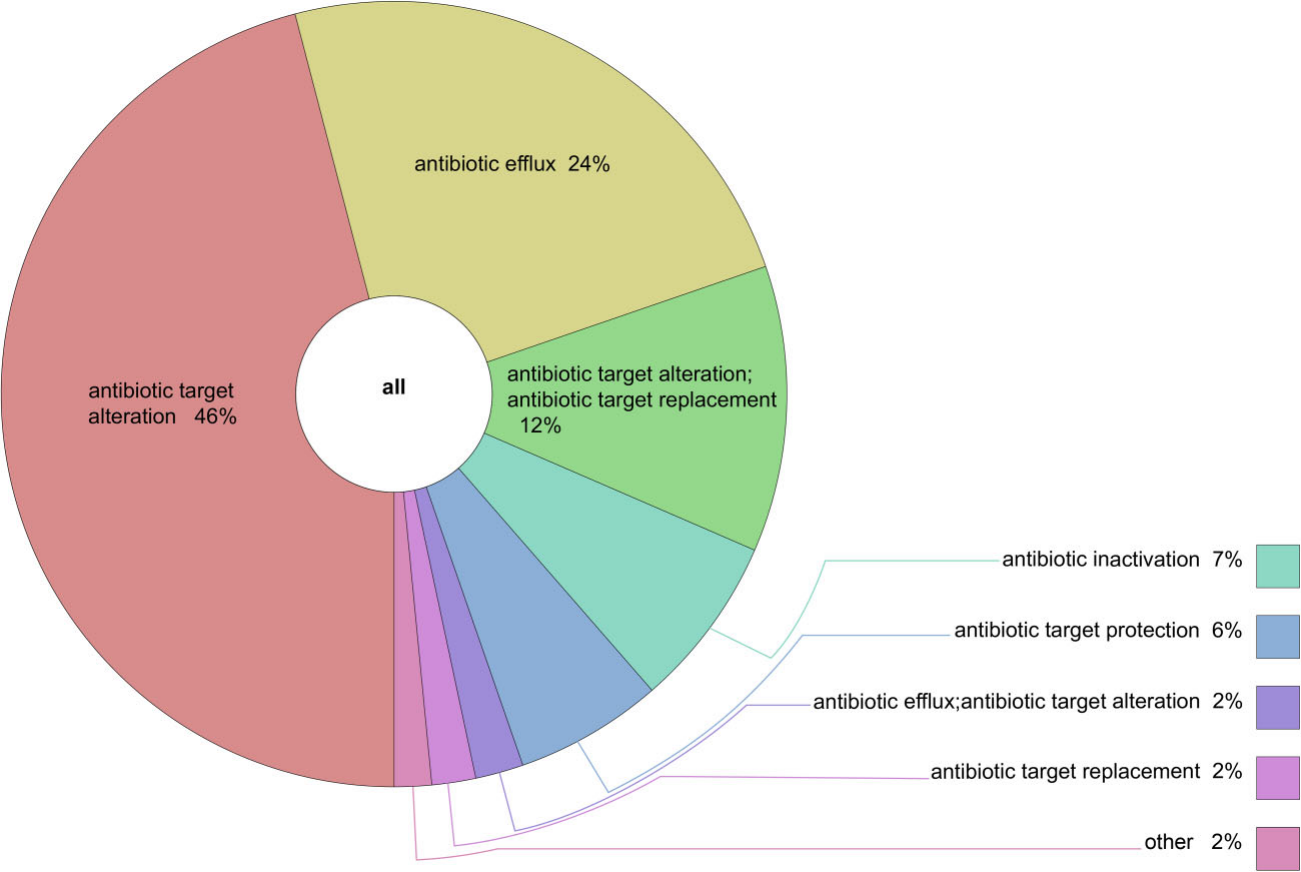
Krona plot showing the distribution of drug resistance mechanisms across the combined sample set. Mechanisms adopted are not equally distributed across the different antibiotic groups with antibiotic target alteration, antibiotic target alteration; antibiotic target replacement, antibiotic efflux predominant, potentially increasing the potential target range.

**Figure 6.**
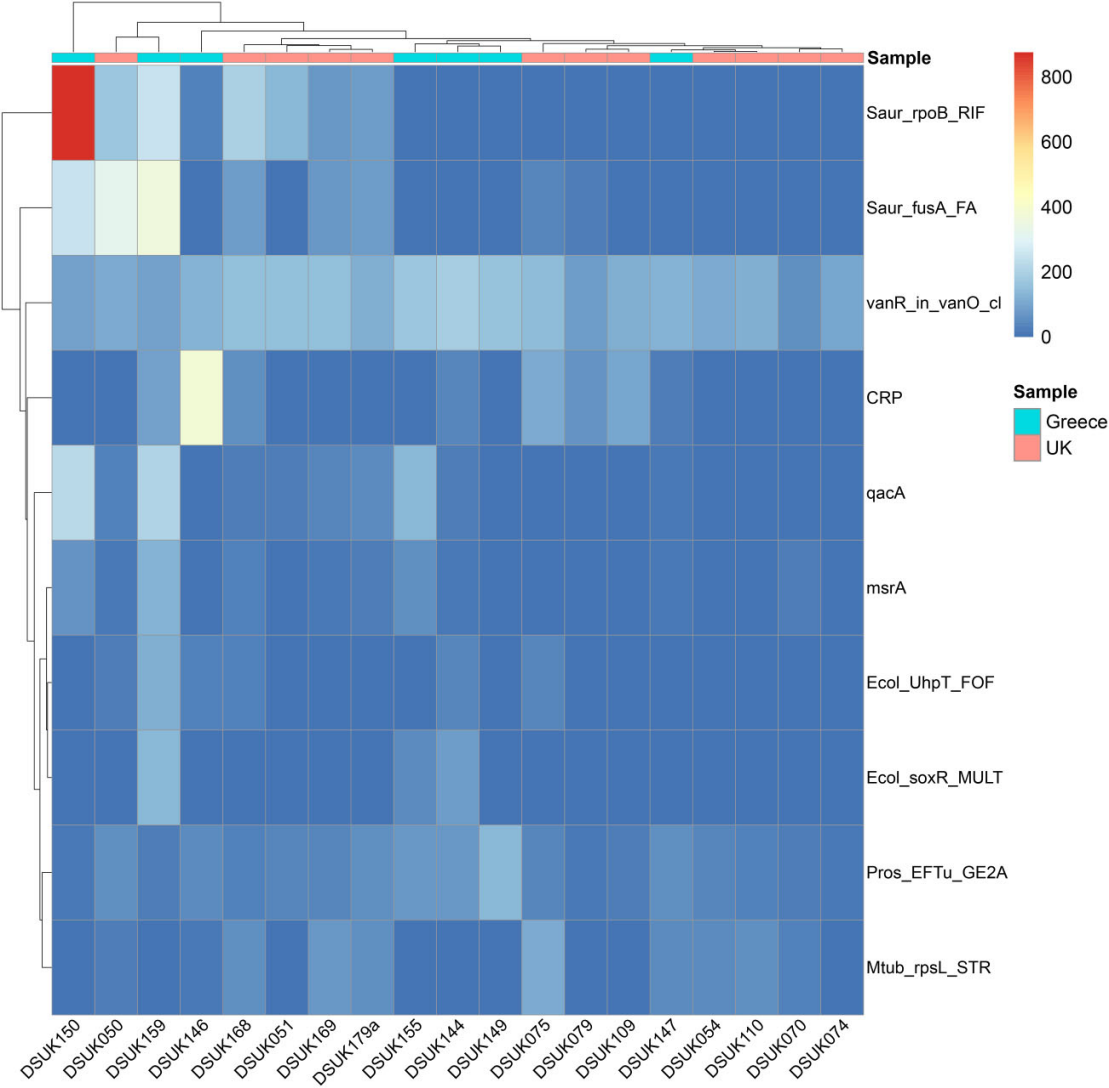
Top 10 AMR gene distribution across all households. The distribution is broad with relatively little in common across different households.

**Figure 7.**
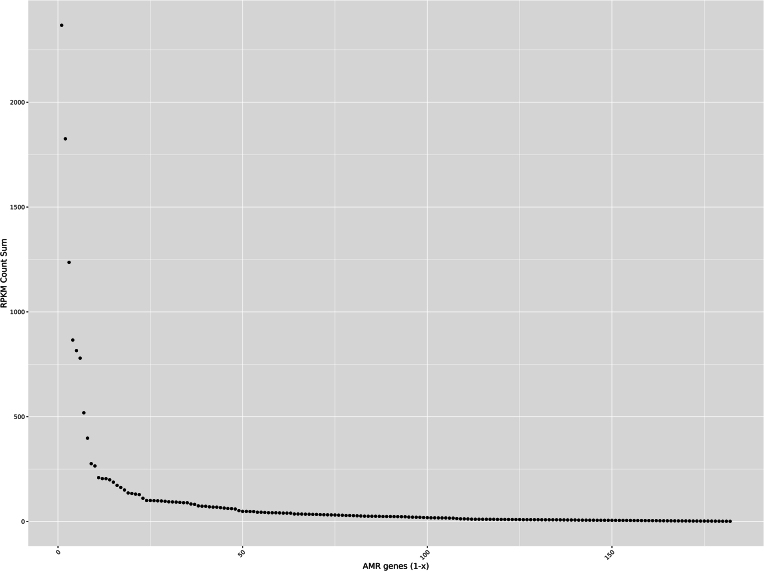
Frequency distribution of AMR genes.

Eleven distinct resistance mechanisms were identified from the detected AMR genes (Table 5). The predominant and most diverse mechanism of AMR in the dust samples were antibiotic target alteration and antibiotic efflux accounting for 70% of genes detected (Fig. 5). The top 10 AMR gene distribution across all households is given in (Fig. 6) where the distribution is broad with relatively little in common across different households. In common with most studies of microbial ecology, the frequency of AMR gene distribution followed a standard long tailed distribution, with a high abundance of the most common AMR gene then a much smaller number of the second, third and so on with a long list of low abundance AMR genes (Fig. 7).

**Table 5.** Resistance Mechanism Table.

Resistance mechanism	Total RPKMs (all samples)	% RPKMs (all samples)
antibiotic target alteration	7267.55	45.95
antibiotic efflux	3761.94	23.78
antibiotic target alteration;antibiotic target replacement	1861.54	11.77
antibiotic inactivation	1122.56	7.10
antibiotic target protection	962.16	6.08
antibiotic efflux;antibiotic target alteration	312.76	1.98
antibiotic target replacement	285.21	1.80
antibiotic efflux;antibiotic target alteration;reduced permeability to antibiotic	100.25	0.63
reduced permeability to antibiotic;resistance by absence	68.28	0.43
reduced permeability to antibiotic	54.50	0.34
antibiotic efflux;reduced permeability to antibiotic	20.19	0.13

## Discussion

### Bacterial species present

The most important sources of microbes in house dust are thought to be outdoor air and other outdoor material tracked into the buildings, occupants of the buildings including pets and microbial growth on moist building materials (Swaebly and Christensen 1952, Hunt and Johnson 2012, Sorkheh et al. 2022). Hence, we might reasonably expect that a proportion of the dust is derived from local soils that move into the house on people's shoes, via pets, or through open windows and ventilation systems (Abramova et al. 2023). However, this appeared not to be the case in this instance. In this study, of the top 50 species identified (Table 2), 30 were predominantly likely to be human commensals or pathogens, and 20 were predominantly likely to be derived from the environment (including soil, plants, rocks, sediments, spider's webs, parasitic nematodes [of non-humans], other animals or food). Of the 50, 14 could also be considered both human and environmental—essentially ubiquitous or cosmopolitan, largely environmental but opportunistically pathogenic (causing infections in very specific or rare cases). Of the ten common genera, six could be considered typical of human studies or human derived material (*Corynebacterium* sp., *Lactobacillus* sp., *Lactococcus* sp., *Salmonella* sp., *Staphylococcus* sp. and *Streptococcus* sp.) and four more although not exclusively, environmental (*Acinetobacter* sp., *Bacillus* sp., *Deinococcus* sp. and *Flavobacterium* sp.). Elsewhere, culture-independent studies have shown estimates of up to 500–1 000 different microbial species in house dust. Concentrations of microbes in house dust vary from non-detectable to 109 cells g-1 dust, depending on the dust type, detection method, type of the indoor environment and season, among other factors, all suggestive of a high degree of heterogeneity and sample by sample variation (Swaebly and Christensen 1952).

### AMR gene overall diversity

The CARD database contains 5 178 AMR genes, meaning the combined samples presented in this study represent 3.5% of the overall AMR genes in the database (182 total AMR genes detected). Several AMR genes detected are ubiquitous and several specific to particular AMR-host associations. However, *vanR* in the *vanO* cluster, a glycopeptide and *Planobispora rosea EF- Tu* mutants conferring resistance to inhibitor GE2270A (elfamycin resistant *EF-Tu*) were the only specific antimicrobial resistance genes found in all samples. Notably, the vancomycin resistance gene *vanA*, a previously suggested indicator of antibiotic resistance contamination of clinical origin, which is thought to be uncommon in the environment was not detected (Tennent et al. 1989). *qacA* occurring in 16/19 samples is major facilitator superfamily (MFS) antibiotic efflux pump and its broad occurrence could be a result of widespread carriage of this gene on *S. aureus* resistance plasmids and hence reflect both the abundance and distribution of this organism in a human environment (Li et al. 2015). *tet(32)* in 15/19 samples is a tetracycline-resistant ribosomal protection protein, a widely distributed gene conferring resistance to tetracycline. Interestingly, the *tetM* gene has been reported to be abundant and has been proposed to be an indicator of the co-occurrence of other ARGs (Zeng et al. 2016). *Erm(O)-lrm* is an Erm 23S ribosomal RNA methyltransferase conferring resistance to lincomycin and macrolide antibiotics and was present in 14/19 samples. *Mycobacterium tuberculosis gyrB* mutant conferring resistance to fluoroquinolones was present in 13 of the 19 samples (Table 6).

**Table 6.** AMR Gene Table.

ARO name	AMR gene family	No. samples	% sam ples	Total RPKMs (all samples)	% RPKMSs (all samples)
*vanR* gene in *vanO* cluster	glycopeptide resistance gene cluster;vanR	19	100.00	2367.27	14.97
*Staphylococcus aureus rpoB* mutants conferring resistance to rifampicin	rifamycin-resistant beta-subunit of RNA polymerase (*rpoB*)	8	42.11	1825.57	11.54
*Staphylococcus aureus fusA* with mutation conferring resistance to fusidic acid	antibiotic resistant *fusA*	8	42.11	1236.21	7.82
*CRP*	resistance-nodulation-cell division (RND) antibiotic efflux pump	8	42.11	865.73	5.47
*Planobispora rosea EF-Tu* mutants conferring resistance to inhibitor GE2270A	elfamycin resistant *EF-Tu*	19	100.00	815.20	5.15
*qacA*	major facilitator superfamily (MFS) antibiotic efflux pump	16	84.21	779.28	4.93
Mycobacterium tuberculosis *rpsL* mutations conferring resistance to Streptomycin	antibiotic-resistant *rpsL*	10	52.63	518.68	3.28
*msrA*	*msr*-type ABC-F protein	12	63.16	397.72	2.51
*Escherichia coli UhpT* with mutation conferring resistance to fosfomycin	antibiotic-resistant *UhpT*	6	31.58	275.88	1.74
*Escherichia coli soxR* with mutation conferring antibiotic resistance	ATP-binding cassette (ABC) antibiotic efflux pump;major facilitator superfamily (MFS) antibiotic efflux pump;resistance-nodulation-cell division (RND) antibiotic efflux pump	3	15.79	265.12	1.68
*mphC*	macrolide phosphotransferase (*MPH*)	10	52.63	209.32	1.32
*norA*	major facilitator superfamily (MFS) antibiotic efflux pump	7	36.84	204.66	1.29
*Escherichia coli parE* conferring resistance to fluoroquinolones	fluoroquinolone resistant *parE*	4	21.05	204.33	1.29
*OXA-496*	OXA beta-lactamase	3	15.79	199.02	1.26
*Erm(O)-lrm*	*Erm 23S* ribosomal RNA methyltransferase	14	73.68	187.78	1.19
*Escherichia coli PtsI* with mutation conferring resistance to fosfomycin	antibiotic-resistant *ptsI* phosphotransferase	7	36.84	172.52	1.09
*tet(C)*	major facilitator superfamily (MFS) antibiotic efflux pump	5	26.32	162.57	1.03
*Escherichia coli gyrB* conferring resistance to aminocoumarin	aminocoumarin resistant *gyrB*	3	15.79	150.43	0.95
*Cutibacterium acnes gyrA* conferring resistance to fluoroquinolones	fluoroquinolone resistant gyrA	4	21.05	136.28	0.86
*dfrC*	trimethoprim resistant dihydrofolate reductase *dfr*	9	47.37	133.85	0.85
*lnuA*	lincosamide nucleotidyltransferase (LNU)	3	15.79	130.72	0.83
*Escherichia coli fabG* mutations conferring resistance to triclosan	antibiotic resistant *fabG*	6	31.58	128.39	0.81
*cmx*	major facilitator superfamily (MFS) antibiotic efflux pump	4	21.05	110.92	0.70
*tet(32)*	tetracycline-resistant ribosomal protection protein	15	78.95	100.33	0.63
*Salmonella serovars soxS* with mutation conferring antibiotic resistance	ATP-binding cassette (ABC) antibiotic efflux pump;General Bacterial Porin with reduced permeability to beta-lactams;major facilitator superfamily (MFS) antibiotic efflux pump;resistance-nodulation-cell division (RND) antibiotic efflux pump	9	47.37	100.25	0.63
*TolC*	ATP-binding cassette (ABC) antibiotic efflux pump;major facilitator superfamily (MFS) antibiotic efflux pump;resistance-nodulation-cell division (RND) antibiotic efflux pump	2	10.53	99.64	0.63
*acrD*	resistance-nodulation-cell division (RND) antibiotic efflux pump	1	5.26	98.84	0.62
*TEM-126*	TEM beta-lactamase	2	10.53	98.06	0.62
*Escherichia coli folP* with mutation conferring resistance to sulfonamides	sulfonamide resistant dihydropteroate synthase *folP*	4	21.05	96.60	0.61
*vgaALC*	*vga*-type ABC-F protein	6	31.58	94.14	0.60
*acrB*	resistance-nodulation-cell division (RND) antibiotic efflux pump	1	5.26	93.55	0.59
*fusB*	Target protecting *FusB*-type protein conferring resistance to Fusidic acid	12	63.16	92.71	0.59
*tet(Q)*	tetracycline-resistant ribosomal protection protein	2	10.53	91.17	0.58
*Mycobacterium tuberculosis gyrB* mutant conferring resistance to fluoroquinolones	fluoroquinolone resistant *gyrB*	13	68.42	89.69	0.57
*lmrD*	ATP-binding cassette (ABC) antibiotic efflux pump	9	47.37	89.51	0.57
*Escherichia coli fabI* mutations conferring resistance to isoniazid and triclosan	antibiotic resistant *fabI*	3	15.79	83.67	0.53
*patA*	ATP-binding cassette (ABC) antibiotic efflux pump	6	31.58	81.89	0.52
*FosD*	fosfomycin thiol transferase	5	26.32	74.73	0.47
*emrB*	major facilitator superfamily (MFS) antibiotic efflux pump	2	10.53	73.13	0.46
*rsmA*	resistance-nodulation-cell division (RND) antibiotic efflux pump	10	52.63	72.82	0.46
*mdtP*	major facilitator superfamily (MFS) antibiotic efflux pump	2	10.53	70.07	0.44
*tet(K)*	major facilitator superfamily (MFS) antibiotic efflux pump	4	21.05	68.86	0.44
*baeR*	resistance-nodulation-cell division (RND) antibiotic efflux pump	1	5.26	68.45	0.43
*Streptococcus pneumoniae PBP2x* conferring resistance to amoxicillin	Penicillin-binding protein mutations conferring resistance to beta-lactam antibiotics	1	5.26	66.21	0.42
*sul1*	sulfonamide resistant *sul*	3	15.79	64.19	0.41
*Staphylococcus aureus gyrB* conferring resistance to aminocoumarin	aminocoumarin resistant *gyrB*	4	21.05	62.27	0.39
*Escherichia coli parC* conferring resistance to fluoroquinolones	fluoroquinolone resistant *parC*	1	5.26	61.51	0.39
*cpxA*	resistance-nodulation-cell division (RND) antibiotic efflux pump	2	10.53	59.64	0.38
*evgS*	major facilitator superfamily (MFS) antibiotic efflux pump;resistance-nodulation-cell division (RND) antibiotic efflux pump	1	5.26	51.87	0.33
*mdtG*	major facilitator superfamily (MFS) antibiotic efflux pump	1	5.26	48.41	0.31
*eptA*	*pmr* phosphoethanolamine transferase	1	5.26	48.41	0.31
*AcrE*	resistance-nodulation-cell division (RND) antibiotic efflux pump	2	10.53	47.81	0.30
*Streptomyces lividans otr(A)*	tetracycline-resistant ribosomal protection protein	11	57.89	47.61	0.30
*Escherichia coli mipA*	*MipA*-interacting Protein	1	5.26	44.30	0.28
*RlmA(II)*	non-erm 23S ribosomal RNA methyltransferase (G748)	5	26.32	44.29	0.28
*pmrA*	major facilitator superfamily (MFS) antibiotic efflux pump	5	26.32	43.24	0.27
*tet(O)*	tetracycline-resistant ribosomal protection protein	1	5.26	41.96	0.27
*qacJ*	small multidrug resistance (SMR) antibiotic efflux pump	6	31.58	41.83	0.26
*ErmX*	Erm 23S ribosomal RNA methyltransferase	5	26.32	41.78	0.26
PC1 beta-lactamase *(blaZ*)	*BlaZ* beta-lactamase	5	26.32	41.14	0.26
*bacA*	undecaprenyl pyrophosphate related proteins	2	10.53	40.21	0.25
*Salmonella enterica gyrA* conferring resistance to fluoroquinolones	fluoroquinolone resistant *gyrA*	6	31.58	40.08	0.25
Plasmid-encoded *cat (pp-cat)*	chloramphenicol acetyltransferase (CAT)	1	5.26	39.90	0.25
*Escherichia coli rpoB* mutants conferring resistance to rifampicin	rifamycin-resistant beta-subunit of RNA polymerase (*rpoB*)	1	5.26	35.98	0.23
*Escherichia coli ompF* with mutation conferring resistance to beta-lactam antibiotics	General Bacterial Porin with reduced permeability to beta-lactams	1	5.26	35.98	0.23
*AAC(6′)-Ib7*	*AAC(6′)*	1	5.26	35.62	0.23
*mdtF*	resistance-nodulation-cell division (RND) antibiotic efflux pump	2	10.53	35.07	0.22
*Escherichia coli murA* with mutation conferring resistance to fosfomycin	antibiotic-resistant *murA* transferase	3	15.79	34.77	0.22
*mecI*	methicillin resistant *PBP2*	3	15.79	34.19	0.22
*rpsJ*	tetracycline-resistant ribosomal protection protein	5	26.32	33.95	0.21
*Staphylococcus aureus walK* with mutation conferring resistance to daptomycin	daptomycin resistant *walK*	1	5.26	33.12	0.21
*mecR1*	methicillin resistant *PBP2*	2	10.53	32.43	0.21
*patB*	ATP-binding cassette (ABC) antibiotic efflux pump	1	5.26	31.99	0.20
*Mycobacterium avium gyrA* with mutation conferring resistance to Fluoroquinolone	fluoroquinolone resistant *gyrA*	4	21.05	31.59	0.20
*oqxB*	resistance-nodulation-cell division (RND) antibiotic efflux pump	1	5.26	31.17	0.20
*Enterobacter cloacae acrA*	resistance-nodulation-cell division (RND) antibiotic efflux pump	3	15.79	30.35	0.19
*Escherichia coli cyaA* with mutation conferring resistance to fosfomycin	antibiotic-resistant *cya* adenylate cyclase	1	5.26	29.97	0.19
*lnuC*	lincosamide nucleotidyltransferase (LNU)	3	15.79	28.81	0.18
*msbA*	ATP-binding cassette (ABC) antibiotic efflux pump	1	5.26	28.79	0.18
*Staphylococcus aureus murA* with mutation conferring resistance to fosfomycin	antibiotic-resistant *murA* transferase	1	5.26	28.09	0.18
*APH(3′')-Ib*	*APH(3′')*	1	5.26	27.40	0.17
*qacG*	small multidrug resistance (SMR) antibiotic efflux pump	3	15.79	26.49	0.17
*aadA16*	*ANT(3′')*	5	26.32	25.61	0.16
*Staphylococcus aureus fusE* with mutation conferring resistance to fusidic acid	antibiotic resistant *fusE*	1	5.26	25.33	0.16
*Escherichia coli nfsA* mutations conferring resistance to nitrofurantoin	antibiotic resistant *nfsA*	1	5.26	25.17	0.16
*YajC*	resistance-nodulation-cell division (RND) antibiotic efflux pump	3	15.79	25.11	0.16
*ErmB*	Erm 23S ribosomal RNA methyltransferase	4	21.05	24.88	0.16
*mphE*	macrolide phosphotransferase (MPH)	1	5.26	23.99	0.15
*Erm(33)*	Erm 23S ribosomal RNA methyltransferase	1	5.26	23.98	0.15
*Escherichia coli LamB*	Sugar Porin (SP)	1	5.26	23.98	0.15
*MAB*	class A Mycobacterium abscessus beta-lactamase	7	36.84	23.65	0.15
*Escherichia coli AcrAB-TolC* with AcrR mutation conferring resistance to ciprofloxacin, tetracycline, and ceftazidime	resistance-nodulation-cell division (RND) antibiotic efflux pump	3	15.79	23.32	0.15
*YojI*	ATP-binding cassette (ABC) antibiotic efflux pump	2	10.53	23.06	0.15
*baeS*	resistance-nodulation-cell division (RND) antibiotic efflux pump	1	5.26	22.82	0.14
*MvaT*	resistance-nodulation-cell division (RND) antibiotic efflux pump	1	5.26	21.30	0.13
*MexF*	resistance-nodulation-cell division (RND) antibiotic efflux pump	1	5.26	20.87	0.13
*smeB*	resistance-nodulation-cell division (RND) antibiotic efflux pump	1	5.26	20.49	0.13
*marA*	General Bacterial Porin with reduced permeability to beta-lactams;resistance-nodulation-cell division (RND) antibiotic efflux pump	2	10.53	20.19	0.13
*AcrS*	resistance-nodulation-cell division (RND) antibiotic efflux pump	1	5.26	19.21	0.12
*LpsA*	Intrinsic peptide antibiotic resistant *Lps*	1	5.26	18.52	0.12
*Staphylococcus aureus UhpT* with mutation conferring resistance to fosfomycin	antibiotic-resistant *UhpT*	1	5.26	17.74	0.11
*qacH*	small multidrug resistance (SMR) antibiotic efflux pump	2	10.53	17.70	0.11
*aadS*	*ANT(6)*	1	5.26	17.29	0.11
*Staphylococcus aureus pgsA* mutations conferringresistance to daptomycin	daptomycin resistant *pgsA*	2	10.53	17.18	0.11
*ACT-35*	ACT beta-lactamase	2	10.53	17.10	0.11
*tet(W)*	tetracycline-resistant ribosomal protection protein	2	10.53	16.26	0.10
*fusD*	Target protecting *FusB*-type protein conferring resistance to Fusidic acid	4	21.05	16.09	0.10
*msrE*	*msr*-type ABC-F protein	3	15.79	14.13	0.09
*ErmF*	Erm 23S ribosomal RNA methyltransferase	2	10.53	12.91	0.08
*ANT(4′)-Ia*	*ANT(4′)*	2	10.53	12.67	0.08
*Escherichia coli uhpA* with mutation conferring resistance to fosfomycin	antibiotic-resistant *UhpT*	2	10.53	12.32	0.08
*catS*	chloramphenicol acetyltransferase (CAT)	1	5.26	11.29	0.07
*Acinetobacter baumannii AbaF*	major facilitator superfamily (MFS) antibiotic efflux pump	1	5.26	10.94	0.07
*Mef(En2)*	major facilitator superfamily (MFS) antibiotic efflux pump	3	15.79	10.92	0.07
*Escherichia coli AcrAB-TolC* with *MarR* mutations conferring resistance to ciprofloxacin and tetracycline	resistance-nodulation-cell division (RND) antibiotic efflux pump	4	21.05	10.83	0.07
*MOX-5*	MOX beta-lactamase	2	10.53	10.75	0.07
*Haemophilus parainfluenzae gyrA* conferring resistance to fluoroquinolones	fluoroquinolone resistant *gyrA*	3	15.79	10.67	0.07
*floR*	major facilitator superfamily (MFS) antibiotic efflux pump	4	21.05	10.65	0.07
*emrK*	major facilitator superfamily (MFS) antibiotic efflux pump	2	10.53	10.14	0.06
*H-NS*	major facilitator superfamily (MFS) antibiotic efflux pump;resistance-nodulation-cell division (RND) antibiotic efflux pump	2	10.53	10.09	0.06
*mecA*	methicillin resistant *PBP2*	1	5.26	9.99	0.06
FusF	Target protecting *FusB*-type protein conferring resistance to Fusidic acid	1	5.26	9.87	0.06
PmrF	*pmr* phosphoethanolamine transferase	1	5.26	9.74	0.06
FosA8	fosfomycin thiol transferase	1	5.26	9.61	0.06
*Mycobacterium abscessus atpE* with mutation conferring resistance to bedaquiline	antibiotic resistant ATP synthase	4	21.05	9.58	0.06
*LpxC*	Acinetobacter mutant *Lpx* gene conferring resistance to colistin	2	10.53	8.91	0.06
*gadX*	resistance-nodulation-cell division (RND) antibiotic efflux pump	2	10.53	8.88	0.06
*MexK*	resistance-nodulation-cell division (RND) antibiotic efflux pump	1	5.26	8.87	0.06
*APH(3′)-Ia*	*APH(3′)*	1	5.26	8.74	0.06
*kdpE*	*kdpDE*	1	5.26	8.69	0.05
*ErmC*	Erm 23S ribosomal RNA methyltransferase	1	5.26	8.39	0.05
*OXA-280*	OXA beta-lactamase	2	10.53	8.33	0.05
*mdtO*	major facilitator superfamily (MFS) antibiotic efflux pump	1	5.26	8.32	0.05
*sul2*	sulfonamide resistant *sul*	1	5.26	8.31	0.05
*gadW*	resistance-nodulation-cell division (RND) antibiotic efflux pump	3	15.79	8.23	0.05
*aadA27*	*ANT(3′')*	1	5.26	7.99	0.05
*cprR*	pmr phosphoethanolamine transferase	2	10.53	7.72	0.05
*mdtM*	major facilitator superfamily (MFS) antibiotic efflux pump	1	5.26	7.45	0.05
*CARB-3*	CARB beta-lactamase	3	15.79	7.31	0.05
*Pseudomonas aeruginosa CpxR*	resistance-nodulation-cell division (RND) antibiotic efflux pump	3	15.79	7.30	0.05
*Escherichia coli emrE*	small multidrug resistance (SMR) antibiotic efflux pump	1	5.26	6.60	0.04
23S rRNA (adenine(2058)-N(6))-methyltransferase*Erm(A)*	Erm 23S ribosomal RNA methyltransferase	1	5.26	6.59	0.04
*mepR*	multidrug and toxic compound extrusion (MATE) transporter	2	10.53	6.44	0.04
*ICR-Mo*	intrinsic colistin resistant phosphoethanolamine transferase	1	5.26	6.27	0.04
*CMH-1*	CMH beta-lactamase	1	5.26	6.24	0.04
*qacEdelta1*	major facilitator superfamily (MFS) antibiotic efflux pump	2	10.53	5.85	0.04
*Acinetobacter baumannii parC* conferring resistance to fluoroquinolones	fluoroquinolone resistant *parC*	1	5.26	5.82	0.04
*Enterococcus faecium liaF* mutant conferring daptomycin resistance	daptomycin resistant *liaF*	1	5.26	5.76	0.04
*Staphylococcus aureus norA*	major facilitator superfamily (MFS) antibiotic efflux pump	1	5.26	5.76	0.04
*tet(39)*	major facilitator superfamily (MFS) antibiotic efflux pump	3	15.79	5.49	0.03
*PER-4*	PER beta-lactamase	1	5.26	5.43	0.03
*mexN*	resistance-nodulation-cell division (RND) antibiotic efflux pump	1	5.26	5.29	0.03
*FosY*	fosfomycin thiol transferase	1	5.26	5.23	0.03
*FosB3*	fosfomycin thiol transferase	1	5.26	5.10	0.03
*Escherichia coli ampC* beta-lactamase	*ampC*-type beta-lactamase	1	5.26	5.05	0.03
*MexB*	resistance-nodulation-cell division (RND) antibiotic efflux pump	1	5.26	4.77	0.03
*Bacillus subtilis rpsE* mutations conferring resistance to spectinomycin	spectinomycin resistant *rpsE*	1	5.26	4.75	0.03
*lmrB*	ATP-binding cassette (ABC) antibiotic efflux pump	1	5.26	4.52	0.03
*APH(6)-Id*	*APH(6)*	2	10.53	4.41	0.03
*tet(X1)*	tetracycline inactivation enzyme	1	5.26	4.27	0.03
*MCR-10.5*	MCR phosphoethanolamine transferase	1	5.26	4.17	0.03
*mdtH*	major facilitator superfamily (MFS) antibiotic efflux pump	1	5.26	4.12	0.03
*SAT-4*	streptothricin acetyltransferase (SAT)	1	5.26	3.69	0.02
*CARB-5*	CARB beta-lactamase	1	5.26	3.58	0.02
*Staphylococcus aureus GlpT* with mutation conferring resistance to fosfomycin	antibiotic-resistant *GlpT*	1	5.26	3.35	0.02
*RbpA*	*RbpA* bacterial RNA polymerase-binding protein	1	5.26	3.33	0.02
*adeK*	resistance-nodulation-cell division (RND) antibiotic efflux pump	1	5.26	3.12	0.02
*FosA*	fosfomycin thiol transferase	1	5.26	2.99	0.02
*QnrB41*	quinolone resistance protein (*qnr*)	1	5.26	2.89	0.02
*tet(38)*	major facilitator superfamily (MFS) antibiotic efflux pump	1	5.26	2.82	0.02
*tetA(46)*	ATP-binding cassette (ABC) antibiotic efflux pump	1	5.26	2.70	0.02
*OXA-296*	OXA beta-lactamase	1	5.26	2.37	0.02
*fosA5*	fosfomycin thiol transferase	1	5.26	2.33	0.01
*Staphylococcus aureus mupA* conferring resistanceto mupirocin	antibiotic-resistant isoleucyl-tRNA synthetase (*ileS*)	1	5.26	2.32	0.01
*sul4*	sulfonamide resistant *sul*	1	5.26	2.25	0.01
*Haemophilus influenzae PBP3* conferring resistance to beta-lactam antibiotics	Penicillin-binding protein mutations conferring resistance to beta-lactam antibiotics	1	5.26	2.25	0.01
*farB*	major facilitator superfamily (MFS) antibiotic efflux pump	1	5.26	2.06	0.01
*nalD*	resistance-nodulation-cell division (RND) antibiotic efflux pump	1	5.26	1.86	0.01
*catB3*	chloramphenicol acetyltransferase (CAT)	1	5.26	1.63	0.01
*Acinetobacter baumannii AbaQ*	major facilitator superfamily (MFS) antibiotic efflux pump	1	5.26	1.44	0.01
*Klebsiella pneumoniae KpnG*	major facilitator superfamily (MFS) antibiotic efflux pump	1	5.26	1.43	0.01
*SAT-2*	streptothricin acetyltransferase (SAT)	1	5.26	1.16	0.01

Sorted by % total RPKMs.

### AMR by potential origin

Given the diversity of AMR genes present, it was not possible to determine the origin of AMR but the identified AMR genes suggested that their origin was likely related to antimicrobials used in human medicine or broad-spectrum antibiotics (up to ∼60%). In this study, among those that represented ≥ 5% of the total, glycopeptide antibiotics accounted for 15% of the total. Perhaps unsurprisingly, peptide antibiotics come next at 12% since these are a chemically diverse class of anti-infective and antitumor antibiotics containing non-protein polypeptide chains (Hancock and Chapple 1999). Third at ∼ 9% was fusidic acid, this is an oral anti-staphylococcal antibiotic that has been used in Europe for more than 40 years to treat skin infections as well as chronic bone and joint infections (Fernandes 2016). Next come the fluoroquinolones (8%) perhaps also unsurprisingly, as they represent a family of broad spectrum, systemic antibacterial agents that have been used widely, so we might expect to find them in the domestic setting. The plasmid-mediated fluoroquinolone resistance gene *qnrS* was also among the most reported genes in a review of soil samples by Abramova and colleagues (Abramova et al. 2023). Elfamycin antibiotic is the final category > 5% and this is used to treat AMR resistant infections.

In a review of the literature covering the last 30 years, Zhuang et al. (2021) cite the most common ARGs from hospitals (multidrug, glycopeptide, and β-lactamide ARGs *mecA, vanA, vanB*, and *bla*) and from farms, wastewater treatment plants, water, and soil (sulfonamide and tetracycline ARGs *sul* and *tet*) (Jian et al. 2021). In six types of habitats (farms, cities, WWTPs, water, soil, and air), the first 50 subtypes of ARGs included eight antibiotic families: β-lactam (*bla*), sulfanilamide (*sul*), tetracycline (*tet*), aminoglycoside (*aad*), multidrug (*mec*), amphenicol (*flo*), trimethopolyl (*dfr*), and glycopeptide (*van*). Among them, *mecA* (multidrug) was the most common in the world, followed by β-lactam (*bla*), glycopeptides (*vanA, vanB*), sulfonamide (*sul1, sul2*), and tetracycline (*tetM*) (Williams et al. 2023).

Few microbial species are actually able to grow and proliferate in dust and only if enough moisture is provided. Hence, most of the microbial content originates from sources other than the dust itself (Swaebly and Christensen 1952). One commonly cited potential sources of house dust and the AMR it contains is soil (Forsberg et al. 2014). Soil is considered a hotspot for antimicrobial-producing bacteria, due to its high complexity and competition between the microorganisms present (Armalytė et al. 2019). When the resistome of the soils was assessed, a low variety of resistance determinants was detected (resistance to β-lactams, aminoglycosides, tetracycline, erythromycin, and rifampicin—all are used in human and veterinary medicine) (Mikulic 2024). The same soil samples were also used to isolate antibiotic-resistant cultivable bacteria, which were predominated by highly resistant isolates of P*seudomonas, Stenotrophomonas, Sphingobacterium* and *Chryseobacterium* genera.

### Antibiotics and their derivatives prescribed in the United Kingdom

The ten leading antibacterial drugs dispensed in England in 2021, by number of items were: Amoxicillin (7 874 302), Nitrofurantoin (4 150 721), Flucloxacillin sodium (3 754 633), Doxycycline hyclate (2 962 900), Phenoxymethylpenicillin (Penicillin V) (1 834 967), Clarithromycin (1 644 328), Trimethoprim (1 495 305), Metronidazole (1 353, 724), Co-

amoxyclav (Amoxicillin clavuanic acid) (1 307 794), Lymecycline (1 020 600) (Nowakowska et al. 2019). The 33 most commonly prescribed antibiotics in the UK are amoxicillin, ampicillin, azithromycin, cefaclor, cefadroxil, cefalexin, cefazolin, cefixime, cefoxitin, cefprozil, cefradine, ceftazidime, ceftriaxone, cefuroxime, cephalosporins, chloramphenicol sodium succinate, ciprofloxacin, clarithromycin, coamoxiclav, co-amoxiclav, doxycycline, erythromycin, flucloxacillin, fosfomycin, levofloxacin, moxifloxacin, neomycin, nitrofurantoin, norfloxacin, ofloxacin, phenoxymethylpenicillin, pivampicillin and trimethoprim (Dolk et al. 2018). Others include oxytetracycline; tetracyclines; extended-spectrum penicillins; β-lactamase-sensitive penicillins; β-lactamase- resistant penicillins; combinations of penicillins; macrolides; fluoroquinolones; imidazole, nitrofurantoin (Miyakis et al. 2011). Of these antibiotics, AMR to tetracyclines, fluoroquinolones, macrolides, β- lactams, fosfomycin, chloramphenicol and penicillin were encountered in more than one household, indicating the potential for a link between antibiotics that the house microbiome are exposed to and a human medical treatment origin.

### Antibiotics and their derivatives prescribed in the Greece

The most frequently prescribed antibiotics in Greece were macrolides (29.9%), second and third generation cephalosporins (26.9%), fluoroquinolones (21.0%), and penicillins (15.4%, a quarter of which were narrow spectrum). Clarithromycin (19.8%), cefuroxime (11.8%), ciprofloxacin (11.6%), amoxicillin/clavulanic acid (10.8%) and cefprozil (7.6%) made up 61.6% of all prescribed antibiotics. Trimethoprim/sulfamethoxazole was rarely prescribed, accounting for 1.6% of total prescriptions (Kourlaba et al. 2016, Kopsidas et al. 2023 and European Centre For Disease Prevention and Control 2023).

### Frequency of AMR genes in other bacterial populations

In a global baseline for qPCR-determined antimicrobial resistance gene prevalence across environments, Abramova et al. (2023) collected data that included 1 594 samples distributed across 30 different countries and 12 sample types, in a time span from 2001 to 2020. They found that for most ARGs, the typically reported abundances in human impacted environments corresponded to one ARG copy in a thousand bacteria. An average of 7 000 bacterial species have been reported in house dust from 1 200 homes in the USA. Genome sequences showed that parasitic bacteria have 500–1 200 genes and free-living bacteria have 1 500–7 500 genes per genome, so this would represent a total of 3.5 M to 52.5 M microbial genes (Brocchieri 2021), hence 3 500–52 500 ARGs in total.

Typical environmental studies report 23–477 AMR genes, for example, large-scale commercial swine farms in China 149 (Zhu et al. 2013), park soils 147 (Wang et al. 2014), South-Western highlands of Saudi Arabia 102 (Yasir 2022), Ba River, China 23 (Jia et al. 2021), ready to eat food 477 (Haynes 2021), Antarctic soil 117 (Van Goethem et al. 2018, Hwengwere et al. 2022). In this study 182 AMR genes (15 817 RPKMs total across all samples and AMR genes) from 80 CARD AMR Gene families were identified.

#### AMR gene frequency by mode of activity

The pattern seen in the mode of action data appeared to be that the more general the activity, the more frequently it occurred. The five core mechanisms of AMR are inactivation or modification (enzymatic degradation of antibacterial agents), alteration of target- or binding site (alteration of bacterial proteins that are antimicrobial targets or alteration of metabolic pathway, ribosome splitting and recycling), acquisition of a target by-pass system, reduced drug accumulation (changes in membrane permeability to antibiotics and removal from the cell or increasing active efflux). In this study, we found antibiotic target alteration > antibiotic efflux > antibiotic target alteration or antibiotic target replacement > antibiotic inactivation > antibiotic target protection > antibiotic efflux and antibiotic target alteration > antibiotic target replacement > antibiotic efflux, antibiotic target alteration and reduced permeability to antibiotics > reduced permeability to antibiotics and resistance by absence > reduced permeability to antibiotics > antibiotic efflux and reduced permeability to antibiotic.

Given that the top five mechanisms were represented across samples and that antibiotic target alteration and antibiotic efflux, perhaps unsurprisingly, were the most diverse mechanisms (since these can be effective against a broader range of potential targets), it seems likely that generic rather than specific AMR genes and mechanisms are likely to be important in house dust.

This pattern also appears to occur in other systems, for example, recent soil metagenome studies have shown the relative dominance of determinants encoding bacterial efflux systems compared to other resistance mechanisms such as enzyme-mediated drug modification or drug target binding (Zeng et al. 2026; Hwengwere et al. 2022). In the soil study by Armalytė et al. (2019), the resistance of isolates was largely dependent on the efflux mechanisms, the soil *Pseudomonas* spp. relying mostly on RND, while *Stenotrophomonas* spp. and *Chryseobacterium* spp. on RND and ABC transporters (Mikulic 2024). In the Taihu Lake study (Fernanda et al. 2022), the most dominant mechanism of ARGs was antibiotic inactivation (46%), followed by efflux pump (18%), target alteration (16%), target protection (11%), target replacement (7%), and these results were consistent with a previous study, in which antibiotic deactivation and efflux pump were found to be the most dominant antibiotic resistance mechanisms in bacteria in drinking water treatment systems in China (Wang et al. 2014; UK-VARSS 2023). In Antarctic soil, efflux pump and inactivation were the main mechanisms of resistance (Hwengwere et al. 2022).

### Presence of other animals in households

The proportion of antibiotics proscribed to cats and dogs in the United Kingdom are: Beta- lactams (27%), Tetracyclines (34%), Trimethoprim/sulphonamides (11%), Macrolides (10%), Aminoglycosides (10%), Fluoroquinolones (0.34%), Third and Fourth generation cephalosporins (0.13%), Colistin (0.0002%) and other (Amphenicols, lincomycins, pleuromutilins, steroidal antibiotics and imidazole derivatives) (8%) (Youenou et al. 2015). Many of these, especially the Beta-lactams, Macrolides, Tetracyclines and Fluoroquinolones featured in the AMR detected in house dust in this study.

### Comparison with other studies

Elsewhere, the microbiome and antibiotic resistome have been determined in household dust from Beijing, China (Ding et al. 2020) they also found a great diversity of antibiotic resistance genes in particular to vancomycin and Macrolide-Lincosamide-Streptogramin B. Their resistome profile showed no distinct geographical pattern, and was primarily driven by certain bacterial phyla and occupancy-related factors. Dust samples were also collected from Irish homes in a house dust mite study (Aljohani et al. 2024). They found 176 antimicrobial resistance genes encoding resistance for multi drug resistances, macrolide-lincosamide-streptogramin B, mobile genetic elements, Beta-lactam, Tetracycline and Aminoglycosides. They found no significant difference between ARGs for homes with and without a history of antibiotic use. A pilot study Klavova et al. (2024) aimed to analyze the resistome in indoor dust samples from participants’ workplaces (a pediatric hospital, a maternity hospital, and a research center) and households. In total, 143 ARGs were detected with ARGs associated with the macrolides, lincosamides, and streptogramin B (*MLSB*) being the most abundant, followed by MDR (multi-drug resistance) genes, and genes conferring resistance to aminoglycosides. In a first to look for an association between antibiotic resistance genes and antimicrobial chemicals in dust, Hartmann et al. (2016) found that the three most highly abundant antibiotic resistance genes were *tet(W), blaSRT-1*, and *erm(B)*. In terms of mobilizable antibiotic resistance genes and mobile genetic elements, Maamar et al. (2020) integrated 166 dust metagenomes from 43 different buildings. They found 183 ARGs, of which 52 were potentially mobile (associated with a putative plasmid, transposon or integron), while Feng et al. (2022) found 9 types of ARGs such as sulfonamide, tetracycline, and beta-lactamase and 71 subtypes of ARGs like *sul1, tetM-01*, and *drfA1* were detected in dust from dense urban public places.

### Is there evidence of a core resistome?

Diverse household samples, regardless of bioclimatic region, building type or occupancy, were shown to share a common core microbiome (Thompson et al. 2021). Hence, microbial assemblages in different house dust types usually share the same core species, although differences in the composition can be caused by different sources of inocula for the different dust types. However, in terms of a core resistome, a more restricted pattern emerged whereby only two specific AMR genes were found in every household. Even at the gene family level, only four categories were found in all 19 samples. The next most abundant was found in all but two, the next eight classes were found in more than half the samples (so the top 14 classes were found in more than half of the samples, the bottom 66 were found in less than half of the samples). Importantly, 21 classes were found in only one sample. Hence overall each sample appeared to have its own diverse suite of AMR genes, with little evidence of a core resistome (Figs. 4 –6).

## Summary

This study aimed to investigate whether a core resistome was present in diverse house dust samples. Although each house was different, and AMR gene frequency tended to be sample dependent (with a high degree of variability), common patterns apparently reflected those AMR genes with a broad and widespread pattern of resistance mechanisms. Hence there was no evidence for a core resistome.

A relatively high proportion of the AMR genes detected could potentially be indirectly linked to antibiotic use and the genes identified were not predominantly derived from the external environment. For example, the glycopeptides (15% in our study) were identified as amongst the most common ARGs from hospitals in the last 30 years (Jian et al. 2021) and fluoroquinolones are often proscribed to cats and dogs in the United Kingdom (Youenou et al. 2015).

The results did suggest generic mechanisms might be important in house dust, since the most common mode of action for AMR activity was antibiotic target alteration (to hinder recognition) and antibiotic efflux (to remove the antibiotic), potentially representing a compromise between certainty of working and metabolic energy investment required. However, although a large number of AMR genes were detected across multiple samples, AMR genes could be distributed among 80 CARD AMR gene families (Table 3). Although the top 4 gene families were found across all samples, there was a high degree of heterogeneity across different dust samples in terms of actual gene composition and 25 gene families occurred in only one sample, suggesting a much larger sample size would be necessary to look for clear patterns and to link them with specific features of each household.

## Supplementary Material

qvaf022_Supplemental_File

## Data Availability

Supplementary information is contained in Supplementary Table S1 and S2 and online ENA accession for the study is PRJEB49546 (currently private but will be released upon acceptance).
