# Supplementary material for: House dust—a Pandora's box of antimicrobial resistance (AMR) activity?
Source: Sustain Microbiol. 2025 Sep 16;2(4):qvaf022. doi: 10.1093/sumbio/qvaf022 (PMC13375292; doi:10.1093/sumbio/qvaf022)
Supplement: Supplementary file 1 [file qvaf022_Supplemental_File.docx]

List of Supporting Material Legends

Table S1: Location and key characteristics of the homes in the dataset, captured via the Home Biome questionnaire.

Table S2: Descriptive statistics highlight the order of magnitude concentration range across each of the trace elements (with the exception of Mn) across the 19 samples.

Table S3: Read Summary Table

Table S1: Location and key characteristics of the homes in the dataset, captured via the Home Biome questionnaire

| **Sample ID** | **Location (Town/City)** | **Season of sample submission** | **Home Type** | **Home fabric** | **Occupant number** | **Male & Female occupants** | **Indoor smoker (yes/no)** | **Pets (yes/no)** | **Vacuum frequency (w/2m)** | **Outdoor vacuum**  **use (c/g/w/p)** | **Home age** | **Primary heating** | **Dominant flooring** | **Garden (yes/no)** |
| --- | --- | --- | --- | --- | --- | --- | --- | --- | --- | --- | --- | --- | --- | --- |
| **UK** |  |  |  |  |  |  |  |  |  |  |  |  |  |  |
| **DSUK50** | Omagh | Summer | D | brick | 2 | 1 F & 1 M | N | N | w | p | <40 y | Oil | Wood | Y |
| **DSUK51** | Omagh | Summer | D | brick | 2 | 1 F & 1 M | N | N | w | p | <40 y | Oil | Wood | Y |
| **DSUK54** | Omagh | Summer | D | Stone/cement | 4 | 1 M & 3F | N | Y | w | c,g,w,p | >100 | Oil | Wood | Y |
| **DSUK70** | Newcastle upon Tyne | Autumn | SD | brick | 6 | 2 M & 4 F | N | N | w | c | 51-100 | Gas | Carpet | Y |
| **DSUK74** | Newcastle  upon Tyne | Autumn | SD | brick & timber | 3 | 1 M & 2 F | N | N | 2m |  | 51-100 | Gas | Wood | Y |
| **DSUK75** | Newcastle  upon Tyne | Autumn | SD | brick | 4 | 2 M & 2 F | N | Y | w |  | 51-100 | Gas | Carpet | Y |
| **DSUK79** | Newcastle  Upon Tyne | Autumn | SD | brick | 4 | 3 M & 1 F | N | Y | w |  | 51-100 | Gas | Carpet | Y |

| **DSUK109** | Newcastle  upon Tyne | Autumn | SD | brick & timber | 1 | 1 M | N | N | w | c | 51-100 | Gas | Carpet | Y |
| --- | --- | --- | --- | --- | --- | --- | --- | --- | --- | --- | --- | --- | --- | --- |
| **DSUK110** | Newcastle  upon Tyne | Autumn | SD | brick | 3 | 1 M & 2 F | N | Y | w |  | 51-100 | Gas | Carpet | Y |
| **DSUK168** | Oxford | unknown | SD | brick | 4 | 2 M & 2 F | N | N | w | w | >100 | Gas | Carpet | Y |
| **DSUK169** | Oakham | unknown | SD | brick & stone/cement  & timber | 1 | 1 M | N | N | w | c | 51-100 | Gas | Carpet | N |
| **DSUK179a** | London | unknown | SD | brick | 3 | 2 M & 1 F | Y | Y | w |  | >100 | Gas | Wood | Y |
| **Greece** |  |  |  |  |  |  | N | N |  |  |  |  |  | Y |
| **DSUK144** | Thessaloniki | Summer/  Autumn | D | brick | 4 | 2 M & 2 F | N | N | w |  | <40 y | Gas | Wood | Y |
| **DSUK146** | Thessaloniki | Summer/  Autumn | SD | brick | 4 | 2M, 1F, 1  other | N | N | w |  | <40 y | Other | Tiled | Y |
| **DSUK147** | Thessaloniki | Summer/  Autumn | F | brick | 4 | 1 M & 3F | N | N | w |  | <40 y | Oil | Tiled | N |
| **DSUK149** | Thessaloniki | Summer/  Autumn | F | brick | 3 | 2 M & 1 F | Y | N | w |  | 50-40 y | Gas | Wood | N |
| **DSUK150** | Thessaloniki | Summer/  Autumn | F | brick | 3 | 1 M & 2 F | Y | N | w |  | <40 y | Wood | Tiled | Y |
| **DSUK155** | Athens | Summer/  Autumn | F | brick | 1 | 1 F | N | N | 2m |  | 50-40 y | Gas | Wood | Y |
| **DSUK159** | Thessaloniki | Summer/  Autumn | SD | brick | 5 | 3 M & 2 F | Y | N | w | c | <40 y | Electricity | Carpet | Y |

D= detached freestanding house, SD= semi-detached joined house, F=unit or flat, w= weekly or more, 2m= twice a month,c= car, g=garage, w=workshop, p=porch

Table S2 Descriptive statistics highlight the order of magnitude concentration range across each of the trace elements (with the exception of Mn) across the 19 samples.

Table S3: Read Summary Table

| **Sample** | **raw_seqs** | **qcd_seqs** | **kraken_seqs** |
| --- | --- | --- | --- |
| DSUK050 | 13385066 | 4941186 | 1556086 |
| DSUK051 | 11737049 | 6019717 | 1668351 |
| DSUK054 | 10643011 | 7703064 | 1517605 |
| DSUK070 | 8819407 | 3702268 | 1161882 |
| DSUK074 | 9187734 | 3185630 | 782593 |
| DSUK075 | 8018450 | 3649466 | 1068997 |
| DSUK079 | 12134939 | 7514401 | 1736529 |
| DSUK109 | 9248883 | 1773991 | 387117 |
| DSUK110 | 6405963 | 5281743 | 1444947 |
| DSUK144 | 4218675 | 3524532 | 963893 |
| DSUK146 | 9897004 | 7941902 | 2323878 |
| DSUK147 | 7370440 | 5638574 | 1603906 |
| DSUK149 | 9188018 | 4272577 | 1403557 |
| DSUK150 | 12289774 | 2691432 | 921471 |
| DSUK155 | 10363158 | 5240733 | 794465 |
| DSUK159 | 10926956 | 4419798 | 1685594 |
| DSUK168 | 8415199 | 4235796 | 1369226 |
| DSUK169 | 8198904 | 5088731 | 1625620 |
| DSUK179a | 6957334 | 4075161 | 1341763 |
| REANEG | 122203 | 85643 | 75505 |
